# The dark side of the black caiman: Shedding light on species dietary ecology and movement in Agami Pond, French Guiana

**DOI:** 10.1371/journal.pone.0217239

**Published:** 2019-06-24

**Authors:** Stephane Caut, Vincent Francois, Matthieu Bacques, Daniel Guiral, Jérémy Lemaire, Gilles Lepoint, Olivier Marquis, Nicolas Sturaro

**Affiliations:** 1 CSIC (Consejo Superior de Investigaciones Cientificas) Departamento de Etologia y Conservacion de la Biodiversidad, Estacion Biologica de Doñana, Sevilla, España; 2 ANIMAVEG Conservation, Villejuif, France; 3 Alligator Bay, Beauvoir, France; 4 IRD (Institut de Recherche pour le Développement), IMBE (Institut Méditerranéen de Biodiversité et d’Ecologie marine et continentale), Aix-Marseille Université, Marseille, France; 5 Centre d’études Biologiques de Chizé, CEBC UMR 7372 CNRS-Université de La Rochelle, Villiers en Bois, France; 6 Laboratory of Oceanology, UR FOCUS, University of Liège, Liège, Belgique; 7 MNHN & Parc Zoologique de Paris, Paris, France; Université de Sherbrooke, CANADA

## Abstract

The black caiman is one of the largest neotropical top predators, which means that it could play a structuring role within swamp ecosystems. However, because of the difficulties inherent to studying black caimans, data are sorely lacking on many aspects of their general biology, natural history, and ecology, especially in French Guiana. We conducted a detailed study of the Agami Pond black caiman population using a multidisciplinary approach. The aim was to better understand the species’ dietary ecology and movements in the pond, and thus its functional role in pond system. We gathered natural history data, tracked caiman movements using satellite transmitters, and characterized feeding ecology via stable isotope analysis. Our study was carried out over three sampling periods and spanned both wet and dry seasons, which differ in their hydrological and ecological conditions. Our results show that black caiman abundance and age demographics differed between seasons in Agami Pond. In the dry season, Agami Pond is one of the only areas within the marsh to hold water. It thus contains large quantities of different fish species, which form the basis of the black caiman’s diet. Caiman body size, a proxy for age class, was around 1.5 meters. During the wet season, which corresponds to the breeding period for migratory birds (e.g., Agami herons), adult black caimans are present in Agami Pond. Adults were most abundant in the inundated forest. There, most individuals measured up to 2 meters. They also exhibited a particular “predatory” behavior near bird nests, preying on fallen chicks and adults. Juveniles and subadults were present during both seasons in the pond’s open waters. These behavioral observations were backed up by stable isotope analysis, which revealed ontogenetic variation in the caiman’s isotopic values. This isotopic variation reflected variation in diet that likely reduced intraspecific competition between adults and young. The telemetry and microchip data show that different age classes had different movement patterns and that seasonal variation in the pond may influence caiman prey availability and reproductive behavior. The new information gathered should help predict this species’ responses to potential ecosystem disturbance (e.g., water pollution, habitat destruction) and inform the development of an effective conservation plan that involves locals and wildlife officials.

## Introduction

Between the estuary of the Amazon River in Brazil and the Cayenne Peninsula in French Guiana lies a series of large mangrove swamps. Of these mostly stagnant wetlands, the Kaw-Roura Marshes are the most distant from the estuary (a biodiversity hot spot; National Nature Reserve 1998—Ramsar 1993). They are located southeast of Cayenne and cover an area of 94,700 ha. Their inaccessibility by road or river has protected them from human disturbance. Within the vast marshes, there are water bodies covered by mats of floating vegetation as well as a few permanent areas of open water—ponds. Among them, one, Agami Pond, is unique in being surrounded by shrub communities [1]. To study this unique and largely uncharacterized ecosystem, a floating scientific platform, only accessible by helicopter, was built in 2001. Preliminary research has since shown that this pond is used by a population of black caimans (*Melanosuchus niger* [2]) and that it is the most important breeding site in French Guiana for many species of birds (e.g., the hoatzin [*Opisthocomus hoatzin*]). Agami Pond also hosts the largest population of the Agami heron (*Agami agamia*): 1,500 breeding pairs [3].

The black caiman has a distributional range that extends from Central America to northern South America. Formerly common, the caiman’s numbers have declined by nearly 99% during the 20th century [4,5], mainly because of strong hunting pressure focused on the animal’s hide. In many areas where this species is still present, older age classes are poorly represented or absent for this same reason [5–10]. The black caiman is one of the largest predators in the Neotropics [4] and is found at the top of the food web. It preys upon species at different trophic levels, which means that it may have a major structuring role within the swamp ecosystem [11].

In French Guiana, the black caiman has been fully protected by law since 1986. The first studies on the Agami Pond population were carried out in 2001, and this preliminary research led to the development of conservation-related research priorities. The results suggest that the Kaw Marsh population (the most northwestern population in French Guiana) is isolated from the much larger ancestral populations in Brazil, and, indeed, the black caiman is rarely seen in other French Guianan coastal wetlands [12]. However, knowledge about its ecological requirements remains scarce [5]. In particular, the lack of information on its trophic ecology, biological interactions, nesting areas, and demography is an important obstacle to the implementation of a long-term conservation program, especially in French Guiana where few scientific studies have been performed. In Brazil, where 80% of the black caiman’s range occurs, scientific and conservation programs based on population monitoring have resulted in an IUCN classification of “Lower Risk” for the species [13–16]. Nonetheless, more in-depth studies are needed to clarify both the species’ conservation status in French Guiana and its role in ecosystem functioning.

To mitigate anthropogenic impacts and optimally delineate protected areas, determining a species’ distribution is paramount. Similarly, characterizing habitat use is vital to understanding animals’ biophysical requirements (e.g., diet, reproduction) and further predicting which locations are of ecological significance [17]. For many species, social conditions influence home range size, and, consequently, population abundance and distribution. To properly manage any species, understanding home range dynamics could be helpful. It is of particular importance, however, when dealing with top predators because of their influence on lower trophic levels [18,19]. Moreover, the black caiman’s habitat, marshes, experiences cyclical variation—wet and dry seasons—which has a determinant influence on water level and plant-animal communities. It is thus likely that prey type and density fluctuate tremendously, forcing top predators to adjust rapidly to survive.

The goal of this study was to describe the black caiman’s dietary ecology and movements in Agami Pond, one of the last sites in French Guinea where adult black caimans are thought to be present. Of particular interest was the key role played by caimans in pond ecology and throughout the entire marsh ecosystem via their interactions with prey communities and given cyclical environmental conditions. Using different approaches (behavioral observations, stable isotope analysis, and telemetry), we conducted a detailed study of the Agami Pond black caiman population to (*i*) examine population age structure and its variation over time and (*ii*) characterize the intra- and interannual movements of individuals and thus identify potential feeding, breeding, and nesting areas within the Kaw marsh system. To this end, we compared results from three sampling periods and across wet and dry seasons, which differ in hydrological and ecological conditions. The new information gathered should help predict this species’ responses to potential ecosystem disturbance (e.g., water pollution, habitat destruction), which is a crucial part of developing an effective conservation plan that involves locals and wildlife officials. Finally, by coupling the stable isotope and telemetry results, we can provide knowledge of future utility to crocodile conservation at the global scale.

## Materials and methods

### Ethics statement

All animal care and use was approved by the Conseil Scientifique Régional du Patrimoine Naturel (CSRPN); La Direction de l'Environnement, de l' Aménagement et du Logement de Guyane (DEAL); and the French Ministry of Ecology and Sustainable Development (N°155/DEAL/2013, N°2015034–0008, N°2014114–007, N°2014114–006, R03-2019-03-14-011), as was the use of the facilities of the Réserve Naturelle de l’Amana. Efforts were made to minimize animal discomfort during sampling, and all animals were released at their site of capture in a timely manner. SC was granted approval to perform animal experimentation (R-45GRETA-F1-04) by the French Ministry of Agriculture.

### Study area

**Fig 1:**
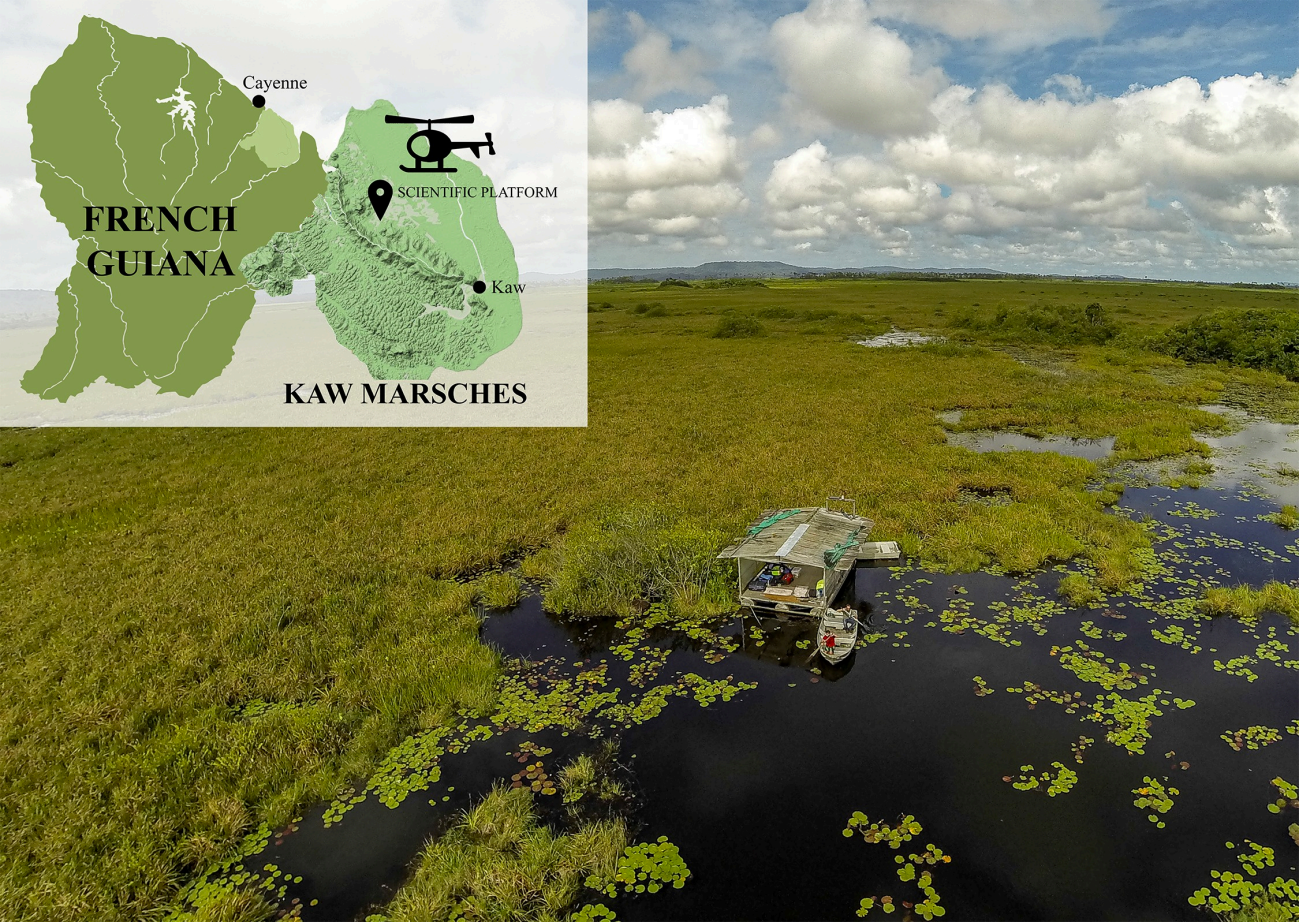
Study site and scientific platform. Location of the Kaw Marshes within French Guiana and picture of the scientific platform (6 x 4 meters) with its two metal boats. Reprinted with permission from S Caut.

The study was conducted in the Kaw-Roura Marshes, a 94,700-ha national reserve in French Guiana (4°36’N, 52°07’W) that is located 40 km southeast of Cayenne (Fig 1). The reserve, which is situated between a coastal mangrove forest on ancient and modern Amazon sedimentary deposit and a continental stunted forest on lateritic cuirass (Kaw mountain, altitude 330 m). It consists principally of waterlogged savannah (Cyperaceae: *Eleocharis interstincta* and *Rhynchospora gigantea*; ferns: *Blechnum serrulatum* and *Thelypteris interrupta*; Araceae: *Montrichia arborescens*) that contains intermittent patches of palm swamp forest (e.g., *Mauritia flexuosa* and *Euterpe oleracea*). Annual rainfall at Kaw mountain (runoff from its northern and western slopes feed the Kaw Marshes) is between 3500 and more than 5000 mm y^-1^. Precipitation is highly seasonal: there is less than 100 mm mo^-1^ in September and November; more than 400 mm mo^-1^ in January, February and June; and more than 700 mm mo^-1^ from April to May [20]. These vast marshes are mostly dominated by grasses and contain water bodies covered by mats of floating vegetation; there are very few permanent areas of open water (i.e., “ponds”). An exception is Agami Pond (04° 38’ N, 52° 09’ W), which is situated in the heart of the Kaw Marshes (Fig 1). Its vegetation is dominated by two fern species (*Thelypteris interrupta* and *Blechnum serrulatum*), which are associated with herbaceous plants (*Sagittaria lancifolia*, *Ludwigia nervosa*, *L*. *torulosa*, *Iribachia alata*, and *Crinum erubescens*) and a rare Orchidaceae (*Habenaria longicaudata*). The pond is locally colonized by highly abundant macrophytes that are either floating and creeping aquatic herbs (*Pontederia rotundifolia*, *Bacopa aquatica*., *Salvinia auriculata*, *Hydrocotyle umbellata*, and *Xyris laxifolia var*. *laxifolia*) or submerged plants (*Cabomba aquatica* and three species of bladderwort: *Utricularia gibba*, *U*. *foliosa*, and *U*. *breviscapa*) associated with rooted species (*Nymphaea rudgeana*, *Nymphoides humboldtianum*, *Eleocharis interstincta*, and *Rhynchospora gigantea*). Discovered in 1998, this pond is inaccessible by foot or boat; people and materials must be transported by helicopter to a floating scientific platform (4 m × 6 m; Fig 1), which was constructed in 2001.

Thanks to preliminary observations from over a decade ago, it is known that Agami Pond seasonally hosts a population of black caimans [12]. Although few data exist, the pond could be an important breeding area. The first surveys of the black caiman in French Guiana were focused on the Kaw River; size-class analysis showed that the proportion of adults was small [21,22].

It is also likely that cyclic environmental conditions in the marsh (wet/dry seasons) could influence prey availability and play an important structural role in this population. Indeed, Agami Pond’s vegetation, which includes an inundated forest patch (*Pterocarpus officinalis*, *Euterpe oleracea*, *Mauritia flexuosa*), provides habitat for nest building by diverse water bird species during the wet season (*Anhinga anhinga*, *Nycticorax nycticorax*, *Egretta alba*, *Cochlearius cochlearius*, *Ardea cocoi*, *Phalacrocorax brasilianus*, *Ophisthocomus hoazin*, and *Agamia agami*; [7]). For this reason, we decided to compare results for the two seasons—wet and dry—which are characterized by different hydrological and ecological conditions. We carried out sampling in Agami Pond during three separate periods: once during the dry season (October 10–17, 2013 [D_1_]) and twice during the wet season (May 1–7, 2014 [W_2_] and May 16–23, 2015 [W_3_]).

### Sampling methods

Using small motorless boats, we surveyed Agami Pond during both seasons to identify potential nesting sites, observe breeding behavior, and quantify biological interactions between the caiman and other species during the day and at night. In addition to this direct data collection, we also gathered indirect data on caiman trophic ecology via the isotopic analysis of multiple tissues; we were thus able to characterize potential variation in feeding location and/or prey.

At night (1900 to 400), we captured caimans from a small motorless boat using a noose. For each individual captured, we first determined total length (TL) and sex. We then implanted a microchip (Virbac) under the skin; these chips helped identify individuals and furnished information on caiman movement between the study seasons. It will continue to provide such data in the years to come. Finally, we sampled muscle and blood for the isotopic analyses. Blood was obtained from the cranial sinus using blood-collection kits (2 ml). The blood sample (1 ml) was immediately separated into red blood cells (RBCs) and plasma by centrifugation. Tail muscle samples were collected from all individuals (Biopunch, diameter of 2 mm, wound closed with a reparative stitch).

To properly conduct the stable isotope analysis, we also needed to know the isotopic values of the caiman’s different potential prey species. To characterize prey availability, we conducted traditional field surveys to identify the major species present. We used fishing methods (nets and rods) to determine fish biodiversity. We first measured length and mass; we then took a sample of dorsal muscle for larger species or collected the entire individual for smaller species. Invertebrates were sampled over three consecutive days using seven pitfall traps (plastic cups with a volume of 20 cm^3^) and two yellow pan traps (20x9x5 cm yellow plastic dishes); the traps were partially filled with soapy water. The pan traps were used to increase the probability of capturing flying insects. This set of traps was placed in different types of terrestrial and aquatic habitats. Newly hatched Agami heron chicks were taken directly from their arboreal nests (sampling period: 0700–1100). Blood samples were obtained from their femoral vein: the vein was punctured at the level of the knee using a sterile 30 gauge needle, and the knee was then bent back and forth in the direction of the chick's body. Each bending movement resulted in a drop of blood. Two to three drops of blood were collected using a heparinized capillary tube. Afterwards, gentle pressure was applied to the wound for 30 sec, and the chick was returned to its nest. Other ecosystem compartments (e.g., plankton and plants) were also sampled to define baseline variation in stable isotopes across time and to compare the ecosystem’s trophic web between seasons and across sampling periods. To sample plankton, we pulled a fine mesh net (50 μm) through the water, either vertically or horizontally, and collected the net’s contents. Plants were sampled by hand; we collected young shoots.

### Stable isotope analysis

Stable isotopes can be used to investigate diet composition, the trophic level of consumers, and even habitat use and migration patterns (e.g., [23–25]). In our analyses, we compared the stable isotope values (carbon and nitrogen) of black caiman tissues (muscle and blood) with those of its potential prey (the species sampled) with the aim of reconstructing its diet. Different groups were identified among the species sampled over the course of the three sampling periods: plants (*Nymphaea rudgeana*, *Pterocarpus officinales*, *Thelypteris interrupta*, *Irlbachia alata*, *Sagittaria lancifolia*, *Chrysobalanus icaco*, *Utricularia hydrocarpa*, *Hydrocotyle umbellata* and 2 Poaceae *sp*.); shrimp (*Macrobrachium jelskii*); amphibians (*Pipa snethlageae*); invertebrates (Aranea, Coleoptera, Diptera, Hymenoptera, Odonata, Orthoptera); birds (*Agamia agami*); and fish, which were divided in four trophic groups: fish^1^ (*Metynnis lippincottianus*), fish^2^ (*Hemigrammus sp*., *Pristella maxillaris*), fish^3^ (*Chaetobranchus flavescens*), and fish^4^ (*Hoplerythrinus unitaeniatu*, *Hoplias malabaricus*).

Samples were dried and ground into a homogenous fine powder. They were then weighed in tin capsules and stored in a desiccator until the analyses took place. These latter were performed using an isotope ratio mass spectrometer (IsoPrime 100, Isoprime, UK) coupled in continuous flow to an elemental analyzer (vario MICRO cube, Elementar, Germany). Stable C and N isotope ratios are conventionally expressed as δ values in terms of %∘ [26]: δ^13^C or δ^15^N = [(*R*_sample_/*R*_standard_)-1]x1000, where *R* is ^13^C/^12^C or ^15^N/^14^N for δ^13^C or δ^15^N, respectively. The certified reference materials from the International Atomic Energy Agency (IAEA, Vienna, Austria) were sucrose (IAEA-CH-6; mean δ^13^C -10.4%∘) and ammonium sulphate (IAEA-N1; mean δ^15^N +0.4%∘). Both of these reference materials were calibrated against the international references Vienna Pee Dee Belemnite for carbon and atmospheric air for nitrogen. Our ten replicate assays of internal laboratory standards indicated maximum measurement errors (SD) of ± 0.2%∘ for both the carbon and nitrogen isotope measurements. Because the tissue C/N ratio was >3.5, we did not correct the δ^13^C values for lipid content [27].

Stable isotope analysis also provides information on food assimilation (and not just ingestion) that is time integrated [28]. One difficulty, however, is that consumer metabolic processes may discriminate between different isotopes. As a result, the consumer’s isotopic ratio may not correspond exactly to the isotopic ratio of the food consumed. This difference between the isotopic composition of an animal’s tissue(s) and its diet is called the discrimination factor, Δ. Moreover, it is imperative to determine the turnover rate, which is the time it takes for the isotope to be assimilated by the consumer. The turnover rates for different tissues must be characterized to assess whether a tissue’s isotopic signature reflects the consumer’s recent diet or long-term diet. To date, only two stable isotope studies focused on basic trophic discrimination dynamics have been performed in crocodilians: Caut [29] looked at the broad-snouted caiman (*Caiman latirostris*), and Rosenblatt and Heithaus [30] researched American alligators (*Alligator mississippiensis*). These studies have shown that turnover rates were slower than those in most of the other taxa studied and that plasma reflected more recent dietary patterns (on the scale of months) than did red blood cells (RBCs) and muscle (on the scale of years). The values of discrimination factors were dependent on several sources of variation (e.g., taxa, site, tissue; see review [31]). Previous laboratory work has shown that significant relationships exist between the δ^13^C and δ^15^N of diets and the Δ^13^C and Δ^15^N of tissues from consumers eating those diets [31, 32]. Using the results of past crocodilian studies [29, 30], we found that these relationships also held true in crocodilians. We thus calculated the diet-dependent discrimination factors associated with each potential prey item using the equations Δ^13^C = -0.03 x δ^13^C -0.98, R^2^ = 0.32 and Δ^15^N = -0.17 x δ^15^N + 0.79, R^2^ = 0.75.

Different isotopic models have been employed to reconstruct animal diets using the isotopic ratios observed in bodily tissues (e.g., see reviews [33, 34]). However, quantitative mixing models are problematic because of their poor resolution of resource mixing space geometry and their poor performance when there are a relatively large number of potential prey. Moreover, because the values of δ^13^C and δ^15^N discrimination factors remain relatively uncertain for crocodilians (and especially for large individuals), mixing models will lead to inconclusive results [31, 33–35], especially without accurate traditional dietary analysis. Consequently, we decided to examine the trophic position of the black caiman relative to other potential ecosystem compartments of importance. However, to perform a quality comparison, the isotopic values of potential prey have been corrected by diet-dependent discrimination factors.

### Satellite telemetry

**Fig 2:**
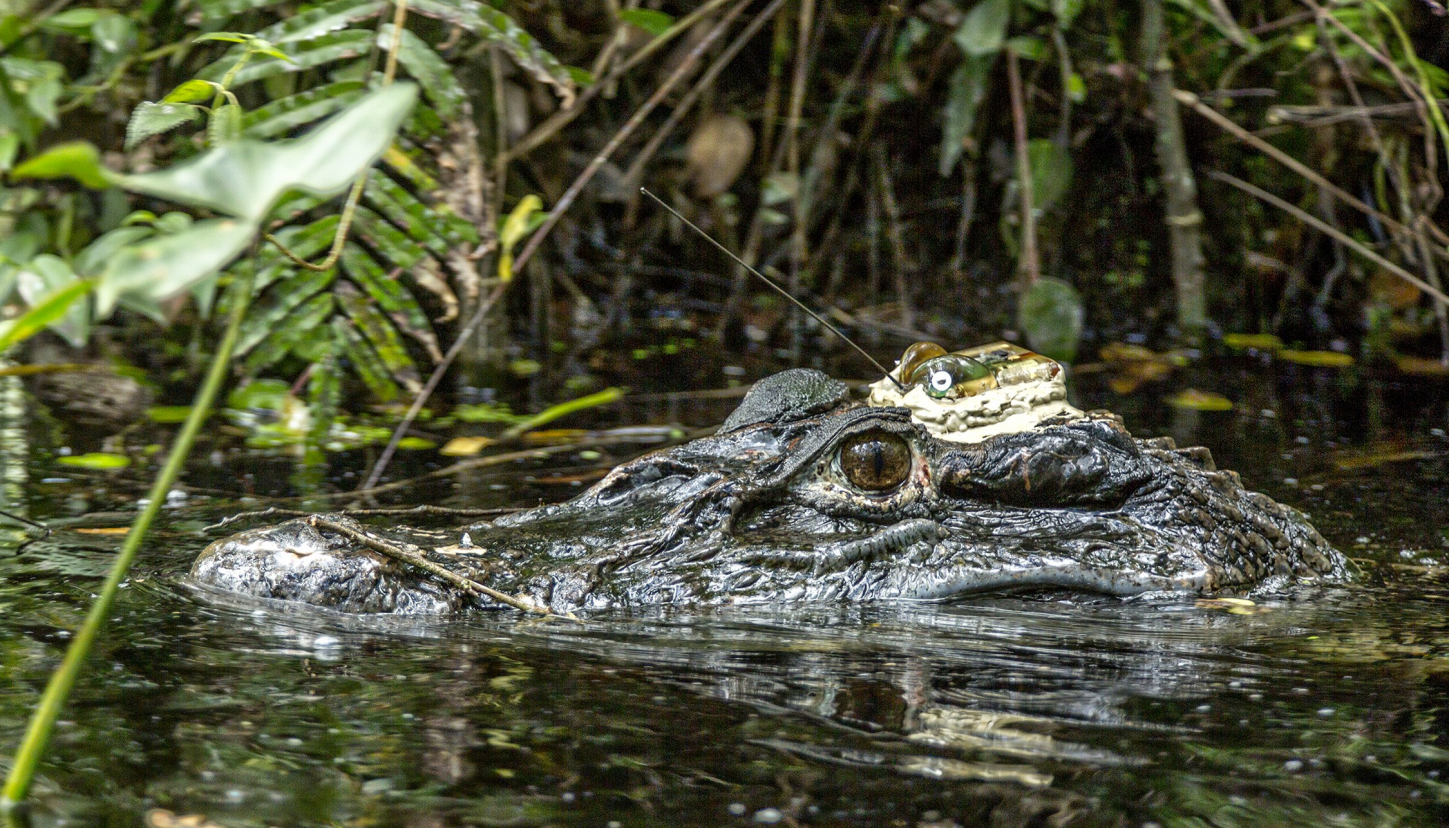
Satellite transmitter attached to a black caiman’s head. Photograph of a male black caiman (*Melanosuchus niger*; M2; TL = 3.2 m) with an Argos satellite transmitter glued to the front of its head. Reprinted with permission from S Caut.

We placed Argos satellite transmitters on adult male and female caimans in May 2015. Given the cost of the transmitters and the challenging field conditions (e.g., efforts were carried out using a 4-m motorless boat due to the abundant floating vegetation), we could only tag four individuals (2.8–3.5 m) (Table 1; Fig 1). The caimans were actively captured using the methods described above; they were restrained while we measured them and fitted them with the transmitters, which were attached to their heads (Fig 2). The front of the caiman’s head was first cleaned with acetone and then lightly sanded (with sandpaper) to improve attachment efficacy. The transmitters were then attached using quick-setting epoxy (Araldite AW 2101 + Durcisseur HW 2951, Gaches, Toulouse, France). We smoothed out the epoxy to provide a streamlined shape. Attaching the transmitters took around 2 h, after which the caiman was allowed to return to the marsh.

**Table 1:** Summary statistics describing the movement patterns of the one female (F1) and three male (M1-M3) black caimans fitted with satellite transmitters. Length (meter) = the total length of the caiman; Tracking days = the total days of GPS tracking; GPS points = the total number of GPS localisation points obtained with four satellites over the tracking days; Distance (meter) = Total distance travelled by the caiman over the tracking days; KUD (meter^2^) = the 95% and 50% volume contours of the kernel utilisation distribution.

Caiman ID	Length (m)	Tracking days	GPS points	Distance (m)	KUD 95% (m^2^)	KUD 50% (m^2^)
**F1**	2.54	341	217	1838	336	69
**M1**	2.72	219	136	1780	21511	3930
**M2**	3.20	357	192	3385	17047	2451
**M3**	3.46	353	162	4128	9984	1915

The transmitters (model SPLASH10-309F; Wildlife Computers Inc., Redmond, WA, USA) relayed Fastloc-GPS data via the Argos satellite system (http://www.argos-system.org). The overall dimensions for each transmitter were as follows: 76 mm in length, 56 mm in width, 32 mm in height, and 125 g in mass (Fig 2). The transmitters were embedded in epoxy resin; their flexible antennae stuck out of the back of the resin blocks at a 45-degree angle. They were programmed to record the caiman’s location once every 15 min during the hours when satellites were passing through the area and to only allow recordings every two days to maximize battery life. However, actual recording frequency was less, presumably because the caimans surfaced intermittently. Only locations associated with a minimum of four satellites and that were accurate to within <250 m were used (Argos User Manual, 2016) [36].

### Statistical analysis

In the isotopic analyses, individuals were grouped into age classes: A (neonates; x<0.50 m), B (juveniles; 0.50≤x<1.2m), C (subadults; 1.2≤x<2 m), and D (adults; x>2 m) [37] (we used total length [TL] because all individuals have completed tails). In our analyses, we used the caiman muscle and blood samples, samples of potential prey, and ecosystem baseline samples (plankton and plants). We compared the results from the October and May sampling periods to contrast trophic status during and outside the bird-breeding season. We first tested whether caimans varied in their isotopic ratios between the wet and dry seasons. We performed factorial ANOVAs to test the effect of season on the isotopic values of each tissue type. The dependent variable was either δ^13^C or δ^15^N, depending on the analysis, and the independent variables were tissue type (muscle, plasma, and RBCs) and season (dry [D_1_] and wet [W_2_ and W_3_]). To determine whether caiman carbon and nitrogen isotopic values differed based on tissue type (muscle, plasma, and RBCs) and age class, we used general linear models (GLMs) in which the dependent variable was δ^13^C or δ^15^N and the independent variables were tissue type and age class. To investigate whether relationships existed between δ^13^C or δ^15^N and age class in each tissue type, we performed general regression models (simple or polynomial). The best fit (simple or polynomial) was chosen on the basis of the regression coefficient (R^2^) and the F and P statistics associated with the full model. Finally, we tested whether the isotopic values of different ecosystem compartments were similar across seasons. We performed one-way ANOVAs where the δ^13^C or δ^15^N of each compartment was the dependent variable, and the season was the independent variable (D_1_, W_2_, W_3_). The normality and homogeneity of all the dependent variables was verified before fitting the models. The statistical analyses were carried out using Statistica v. 6.0 (StatSoft Inc. 2001) and the SAS package (MIXED procedure, v. 8.2, SAS Institute Inc. 2004).

To assess home range size, we employed the fixed kernel (FK) method [38]. For each individual, the 95% and 50% volume contours of the kernel utilisation distribution, or KUD (hereafter, KUD 95% and KUD 50%, respectively) were determined using the “adehabitatHR” package [39] implemented in R software [40].

## Results and discussion

At the beginning of our study, we had no precise information about the Agami Pond population—only reports that this area was of great interest because of the presence of reproductive adults [11]. Our three sampling trips to Agami Pond confirmed that this area is indeed home to a large population. We captured 76 black caimans in total (75 were sampled; Table 2): all were new individuals (i.e., there were no recaptures based on the absence of microchipped individuals). Most measured between 51 cm and 200 cm in total length (age class B = 35.5% and C = 43.5%). It is unclear if this pattern is attributable to the presence of greater numbers of caimans in these age classes or to sampling bias (i.e., individuals in these age classes were easiest to capture); it is likely that it is a combination of both. However, we did observe differences in the age class distribution between seasons. Reproductive adults (age class D) were observed almost exclusively during the wet season and especially in the inundated forest, while juveniles and subadults (age classes B and C) were present during both seasons in the pond’s open waters. Only three individuals measuring less than 50 cm (age class A) were captured over the course of the whole study; they were present exclusively during the wet season. These results—the absence of recaptures, the seasonal abundance of reproductive adults, and the very low numbers of neonates—could suggest that caimans may move large distances (i.e., at least a few km) among years to feed and/or reproduce.

**Table 2:** Number of black caimans sampled by age class (A, B, C, D) for the three sampling periods (D_1_, W_2_, W_3_). *This individual was caught but not sampled because it was too small (TL = 39 cm).

Sampling period	A	B	C	D	Total
**D**_**1**_	0	7	18	1	26
**W**_**2**_	2	7	12	7	28
**W**_**3**_	1*	13	3	5	22

### Dietary differences between seasons and among age classes

**Fig 3:**
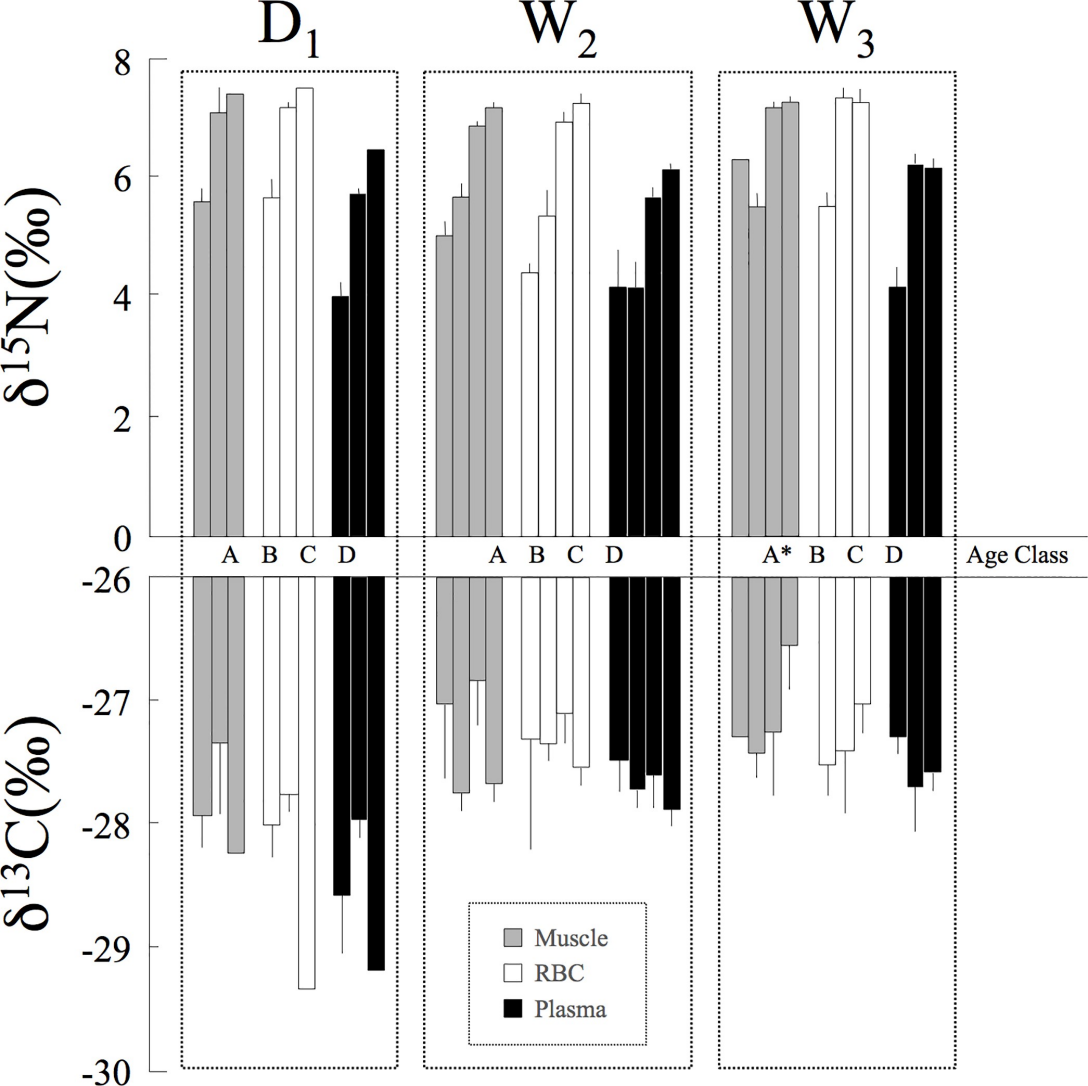
Mean (+SE) δ^15^N and δ^13^C values for black caimans in different age classes. Values are represented for the three sampling periods (D_1_, W_2_, W_3_) and the three tissues sampled (gray = muscle, white = RBCs, and black = plasma). The age classes are represented in the same order for each sampling period (A, B, C, D, with the number of caimans sampled). *During W_3_, only muscle was sampled for the sole individual captured in age class A.

Stable isotope analysis has become an advantageous and complementary tool when characterizing feeding or migration behaviors that are difficult to examine using conventional techniques (e.g., gastric lavage, fecal analysis). It also provides information on the foods that are assimilated, and not just ingested, as well as clarification on how the diet is integrated into tissues over time. To examine seasonal variation in the feeding ecology of the black caiman, it was crucial to sample the different ecosystem compartments during both the wet and dry seasons. The main potential prey items that we caught and sampled were generally the same in both seasons, although the relative importance of water birds and fishes differed. Indeed, the two major seasonal differences were (*i*) the presence of a large breeding colony of aquatic birds, notably the Agami heron, during the wet season and (*ii*) the greater density of fish during the dry season. We found no significant seasonal differences in δ^15^N and δ^13^C for the isotopic baseline or for the individual prey groups (Table 3, S1 Table). These initial results mean that we could compare the isotopic values of juvenile and subadult caimans across seasons (Fig 3). We still found no significant seasonal differences in δ^15^N and δ^13^C; there was, however, a significant difference in δ^15^N among tissues (Table 4, Fig 3). These differences do not necessarily mean that the caiman’s dietary regime varied over time. Instead, they may indicate that the discrimination factor was not the same among tissues. Indeed, for δ^15^N, the interaction between tissue and season was not significant (Table 4). Taken together, these results suggest that the caiman’s diet was similar and stable across seasons for the intermediate age classes (B and C).

**Table 3:** Stable isotope values (δ^13^C and δ^15^N, mean ± SE %∘) for the different ecosystem compartments sampled across seasons (D_1_, W_2_, W_3_). Factorial analysis of variance exploring the effect of season (D_1_, W_2_, W_3_) on the stable carbon and nitrogen isotope values of each compartment. Amphibians were present, but none were caught during the dry season. For the birds, the inundated forest provides habitat for nest building that is used by diverse water bird species and most notably the Agami heron (1,500 breeding pairs). Caimans prey upon fallen chicks and juvenile adults. These potential prey were only present during the wet season.

	Dry (D_1_)			Wet (W_2_)			Wet (W_3_)				δ^15^N		δ^13^C	
Compartments	n	δ^15^N	δ^13^C	n	δ^15^N	δ^13^C	n	δ^15^N	δ^13^C	dfn, dfd	F	P	F	P
**Plankton**	5	0.4 ± 0.2	-30.4 ± 0.5	5	0.5 ± 0.3	-30.1 ± 0.2	5	0.6 ± 0.2	-30.4 ± 0.3	2,12	0.15	0.865	0.26	0.777
**Plant**	10	0.1 ± 0.4	-29.2 ± 0.4	10	0.2 ± 0.5	-29.1 ± 0.5	10	-0.2 ± 0.4	-28.8 ± 0.5	2, 27	2.52	0.097	0.19	0.831
**Invertebrate**	8	4.0 ± 0.2	-26.9 ± 0.4	8	3.6 ± 0.3	-27.3 ± 0.6	7	3.2 ± 0.5	-27.4 ± 0.9	2, 20	1.30	0.294	0.21	0.809
**Shrimp**	5	6.3 ± 0.5	-29.9 ± 0.2	6	5.8 ± 0.2	-29.6 ± 0.3	6	6.3 ± 0.3	-29.2 ± 0.2	2, 14	0.93	0.417	2.33	0.134
**Fish**	17	6.3 ± 0.4	-28.8 ± 0.4	16	6.9 ± 0.5	-29.0 ± 0.4	16	6.3 ± 0.5	-29.0 ± 0.4	2, 46	0.53	0.590	0.14	0.870
**Bird**	* *			18	7.4 ± 0.3	-28.7 ± 0.3	10	7.7 ± 0.1	-29.0 ± 0.2	1, 26	0.45	0.509	0.67	0.422
**Amphibian**	* *			2	8.2 ± 0.3	-28.8 ± 0.6	2	8.5 ± 0.4	-29.1 ± 0.3					

**Table 4:** Variation in black caiman isotopic values. Factorial analysis of variance exploring 1) the effect of season (D_1_, W_2_, W_3_) and tissue type (muscle, plasma, and RBCs) on stable carbon and nitrogen isotope values for black caimans in intermediate age classes (B, C) and 2) the effect of tissue type (muscle, plasma, and RBCs) and total length (TL) on stable carbon and nitrogen isotope values for black caimans in all age classes (A,B,C,D). In italics are the results that were significant in the full model.

				δ^15^N		δ^13^C	
Age class	Variables		dfn, dfd	F	P	F	P
**B**	Season (D_1_,W_2_,W_3_)		2, 72	0.02	0.982	1.22	0.302
** **	Tissue		*2*, *72*	*20*.*84*	*<0*.*001*	0.83	0.441
	Season x Tissue		4, 72	0.23	0.919	1.42	0.236
**C**	Season (D_1_,W_2_)		1, 84	2.59	0.111	0.16	0.690
	Tissue		*2*, *84*	*122*.*02*	*<0*.*001*	*7*.*28*	*0*.*001*
** **	Season x Tissue		2, 84	0.43	0.654	0.48	0.621
**A, B, C, D**	Tissue		*2*, *219*	*16*.*21*	*<0*.*001*	0.07	0.936
	TL		*1*, *219*	*210*.*84*	*<0*.*001*	1.22	0.271
	Tissue x TL		2, 219	0.36	0.702	0.95	0.387

**Fig 4:**
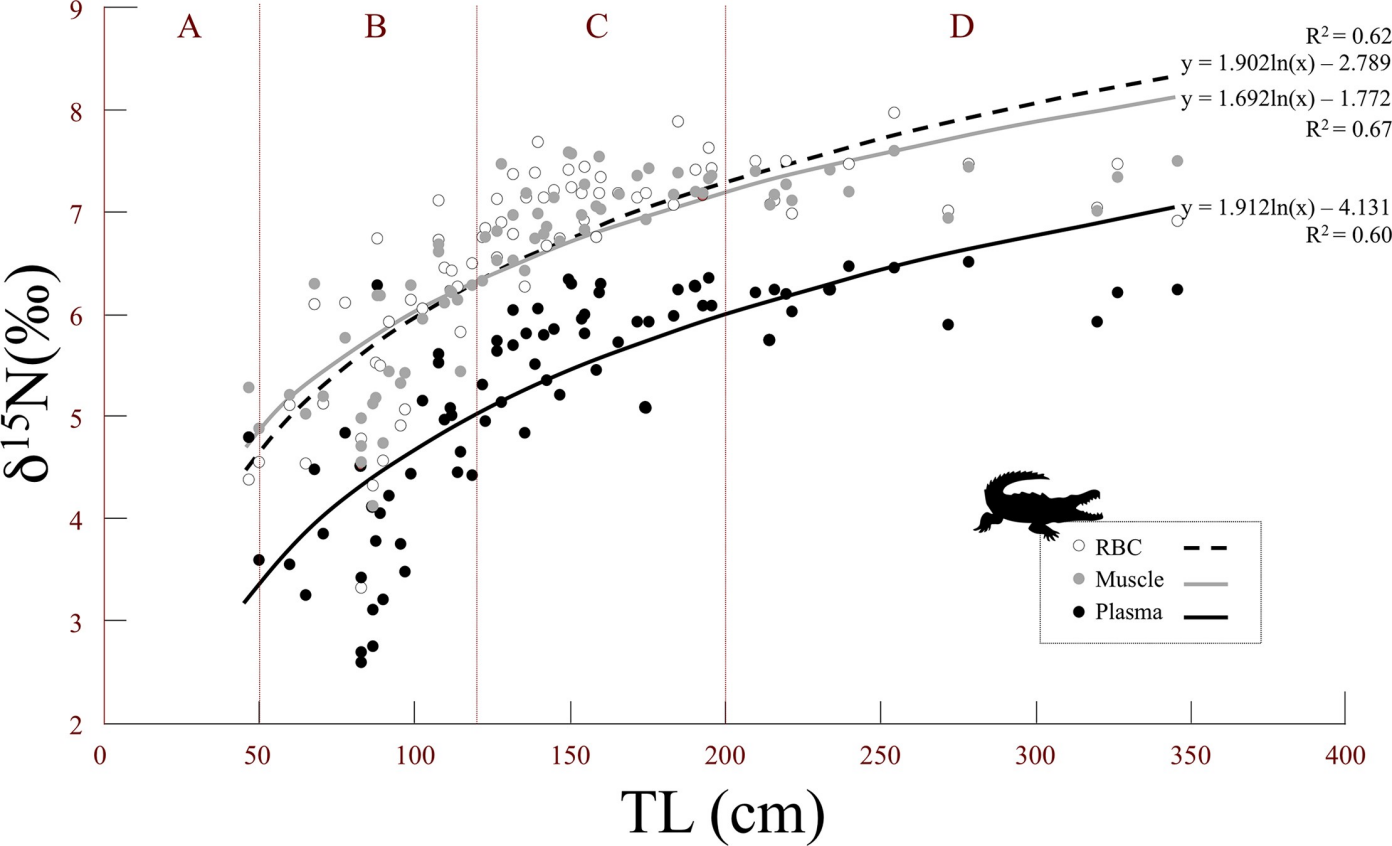
Relationship between black caiman δ^15^N values and total length (TL). Each circle represents one individual (white = RBCs, gray = muscle, and black = plasma). The equation, the coefficient of determination, and the best-fit curve are shown for the significant models (muscle F_1,72_ = 103.80, P < 0.001; plasma F_1,72_ = 69.51, P < 0.001; and RBCs F_1,72_ = 90.43, P < 0.001).

When we took into account all the age classes (A–D), we found a significant effect of size (TL) on δ^15^N (Table 4, Fig 4). This result indicates that size-related differences in resource exploitation exist and that they should consequently reduce competition among age classes [41, 42]. These results confirm the conclusions of research on the Nile crocodile (*Crocodylis niloticus*) [41] and the broad-snouted caiman (*Caiman latirostris*) [43] and also underscore the utility of the stable isotope method for revealing dietary shifts as animals develop. Indeed, crocodilians increase considerably in body size during their lifetimes (1000x from hatchling to adult). Our isotopic results are consistent with the data obtained using traditional diet analyses in this species [44] and other crocodilians [45–47]: over the course of development, there is a dietary shift from invertebrate to vertebrate prey. Juveniles instinctually attack anything that moves on or below the water’s surface (small fish, insects, frogs, molluscs), while adults consume fish, birds, and terrestrial mammals that get too close to the water while walking along the shore; this dietary partitioning appears to reduce intraspecific competition [5,13,42,44,48]. Another interesting hypothesis was explored by Villamarin et al. [49], who looked at isotope patterns in four Amazonian crocodilian species (*Paleosuchus trigonatus*, *P*. *palpebrosus*, *Caiman crocodilus*, and *M*. *niger*). They found that ontogenetic shifts in trophic position revealed by stable isotope analysis may result not only from dietary assimilation but also from trophic discrimination factors (Δ^15^N) associated with body size. Unfortunately, they did not have a large enough sample of large individuals (n = 9 with SVL between 55–110 cm) for the species with the greatest variation in size, *M*. *niger*. These results underscore that, to clarify the trophic ecology of this group, is important to collect isotope data from caimans and their potential prey while also using more traditional methods of dietary analysis, such as observation and assessments of stomach contents.

**Fig 5:**
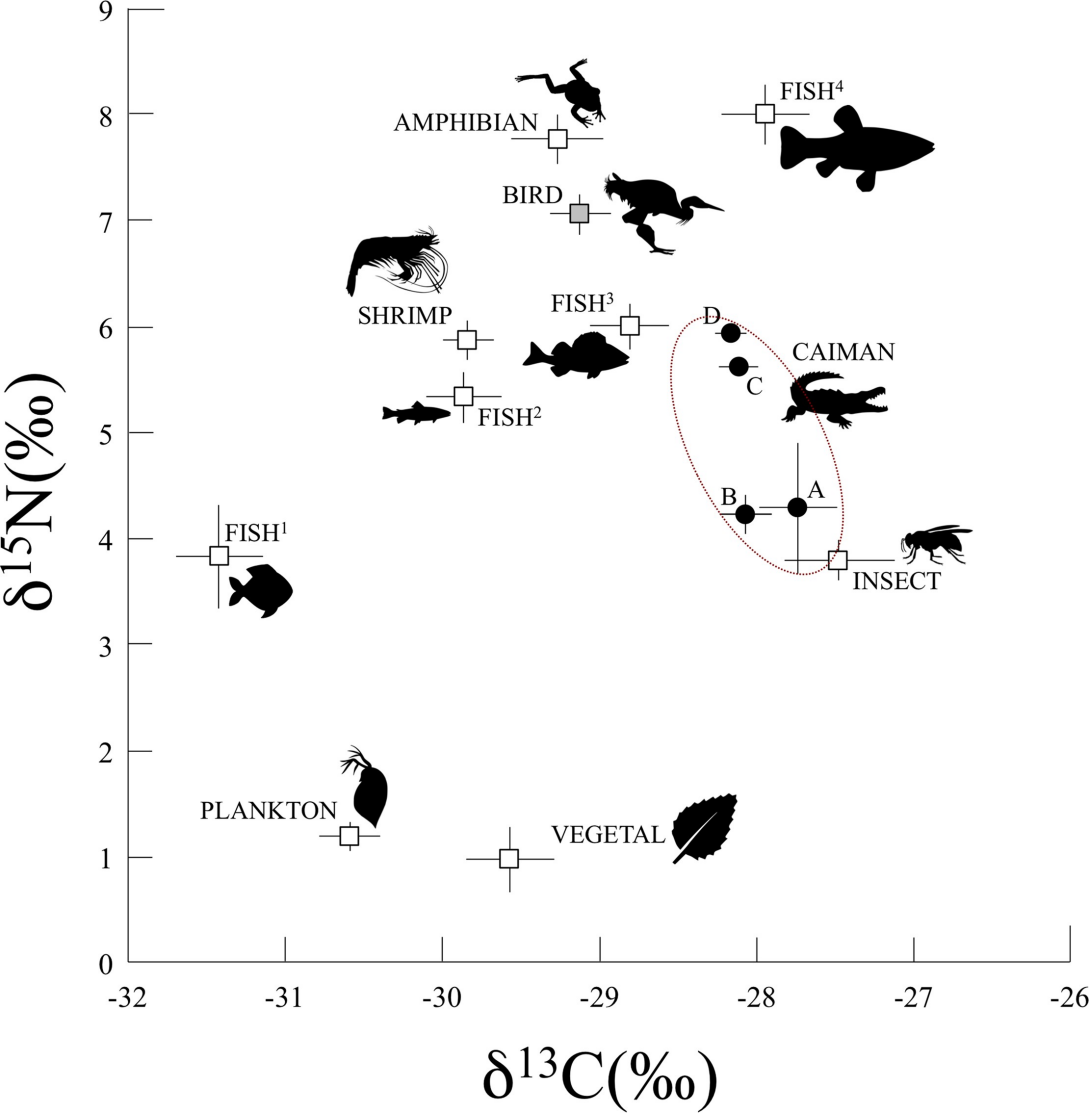
Mean (± SE) δ^15^N and δ^13^C values for the black caimans and the different ecosystem compartments of Agami Pond. The symbols represent each ecosystem compartment. The black circles are the black caimans belonging to different age classes (A, B, C, D); the white squares are the different ecosystem compartments during the wet and dry seasons; and the gray square is the bird compartment (only present during the wet season).

When we compared the isotopic signature of the black caiman’s plasma (corrected for the discrimination factor, see Methods) with those of all the potential prey available in the ecosystem (Fig 5), our findings matched those from previous, more traditional dietary analyses: hatchlings and juveniles occupied a lower trophic position, likely consuming invertebrates and less carnivorous fishes (types 1 and 2), while subadults and adults were at a higher trophic position, likely consuming birds and more carnivorous fishes (types 3 and 4). Given the black caiman’s status as a top predator, it could have had the highest δ^15^N values of any organism in the ecosystem (e.g., adult caiman *vs* carnivorous fish^4^). However, such results are rarely expected, except in the case of specialist top predators. Indeed, the black caiman displays opportunistic behavior and consumes several types of prey at different trophic levels. Consequently, its position in the middle of the trophic web is unsurprising. We nonetheless could have expected to find subadults and adults in a higher position (i.e., closer to the groups that correspond to their potential prey—birds and carnivorous fish^4^). However, it could be that their diet still includes a large proportion of small prey (as mentioned in [44, 50]). It is necessary to interpret these results with caution because a shift from a diet mainly composed of invertebrates to a diet composed of vertebrates would not necessarily cause a linear increase in trophic position. Small crocodilians eat many invertebrates that are predators from high tropic levels, such as mygalomorph spiders, and large crocodilians prey upon vertebrates that may occupy low trophic levels, such as fish and ground-dwelling mammals [49]. Another explanation could be that the discrimination factor was not species or size class appropriate. Animals with large body sizes have tissues with longer isotopic half-lives, and crocodilians have particularly slow turnover rates [49]. Indeed, only two studies have estimated discrimination factors in crocodilians—excretion is very species specific and poorly characterized. In the literature, the ^15^N enrichment of tissues is often attributed to the preferential excretion of light nitrogen (^14^N). However, δ^15^N values vary significantly depending on the form in which nitrogenous waste is excreted. Fasting crocodiles and alligators release approximately equal proportions of ammonia and uric acid in their urine. However, when they are fed ad libitum, the balance shifts: relatively more ammonia is released, and only negligible amounts of urea are produced [45]. Thus, based on what little is known about crocodilians and data obtained from other reptiles, excretion dynamics can affect discrimination factors. This research underscores that experimental data are lacking for isotope incorporation in reptiles and, more precisely, in crocodilians: since 1990, fewer than 10 stable isotope studies have been published on crocodilians, compared to the more than 400 publications available for marine mammals [29,30,41,43,51–54].

Crocodilian social behavior may result in certain individuals having limited access to certain microhabitats, restricting food resources [41,43,44]. In the dry season, Agami Pond is one of the only areas within the marsh that still holds water, resulting in a high abundance of different fish species. They are an important prey item for subadult caimans, which we observed hunting in the open water and at the borders of grass patches. Hatchlings and juveniles were more common at the pond’s edges, where the mat of floating vegetation begins and insects and small fish were quite abundant. The younger caimans consumed these small prey items while avoiding predation by the larger caimans. During the wet season, which corresponds to the breeding period for migratory birds (e.g., Agami herons), adult caimans only occurred in the inundated forest. These large individuals exhibited a very particular "predatory" behavior: in the vicinity of bird nests, they preyed upon fallen chicks and adults. Several black caiman adults were found in the forest, where we observed that they seemed to establish clearly defined areas that they defended aggressively. We twice observed adults attacking juveniles (<1.5 meters) who entered their territory, which sometimes ended in cannibalism. The subadults were therefore forced to remain in the pond’s open water and could not access this seasonal food resource. Indeed, there were no isotopic differences between seasons for these size classes (B and C), whose diets included only fish (i.e., no birds).

The results were more complex for the adults, who were not present during the dry season and who seemed to have a bird-based diet during the wet season. If there was a notable dietary shift, the changes should appeared first in tissues with faster turnover rates (e.g., plasma) versus those with slower turnover rates (muscle and RBCs). We observed a significant relationship between total length and δ^15^N for the three different tissues (Fig 4). In all three cases, the data best fit a quadratic curve, whose form was likely influenced by the tissue-specific discrimination factor. These results suggest that dietary patterns are similar over the short and long term among age classes. For example, if only the adults had displayed a seasonal change in diet, the curves for the muscle and plasma data would not be so similar. Indeed, we would have expected to see a marked difference at the tail end of the curve, reflective of the isotopic values of new prey. However, as explained above, it may also have been that it was difficult to detect dietary shifts between two prey types with similar isotopic values, such as the carnivorous fish (fish^4^) and the birds found in Agami Pond (Fig 5). In the study by Villamarin et al. [55] cited above, carbon isotope values revealed differences among crocodilian species that resulted both from habitat selection and prey preferences. In our case, we found no significant differences in carbon isotope values between seasons and among age classes (Table 4). This result tends to confirm that there were potentially minimal differences in the caimans’ prey type and habitat usage.

Finally, as Laverty et al. [42] noted, the seasonal change in water levels seems to influence prey availability. In Pacaya Samiria (the Peruvian Amazon), caimans avoided the river when water levels were high and were found instead in the shallow waters of the flooded forest. When rivers, other water channels, and lakes are shallower and smaller in area, the animals therein, and especially fish, are easier for crocodilians to catch [13,14]. In our study, the number of captured juvenile and subadult caimans was the same in the open water of the pond regardless of season. We did not observe any evidence of seasonal dietary shifts, although it is important to note that we were not necessarily seeing the same individuals: we did not recapture any microchipped caimans across sampling periods (although we acknowledge that the number of microchipped animals was small). During the dry season, pond water levels were low and fish availability was high [56], but adult caimans were not visible, which seems counterintuitive. Indeed, Laverty et al. [42] found more fish in the stomach contents of the black caiman during periods when water levels were low. When water levels were high and prey availability was thus low, adult caimans were found exclusively in the inundated forest, where they mostly preyed upon birds. This finding suggests that black caimans may change habitat use in relation to prey abundance and occurrence [44]. The seasonality of pond conditions seems to play a key role in ecosystem dynamics and particularly in the feeding behavior of caimans. In addition, it is important to note that the more traditional prey consumed by adult crocodilians—large vertebrates such as anacondas, capybaras, and monkeys [13]—are absent from both the pond and a large proportion of the marshes.

### Satellite telemetry and caiman movement patterns

**Fig 6:**
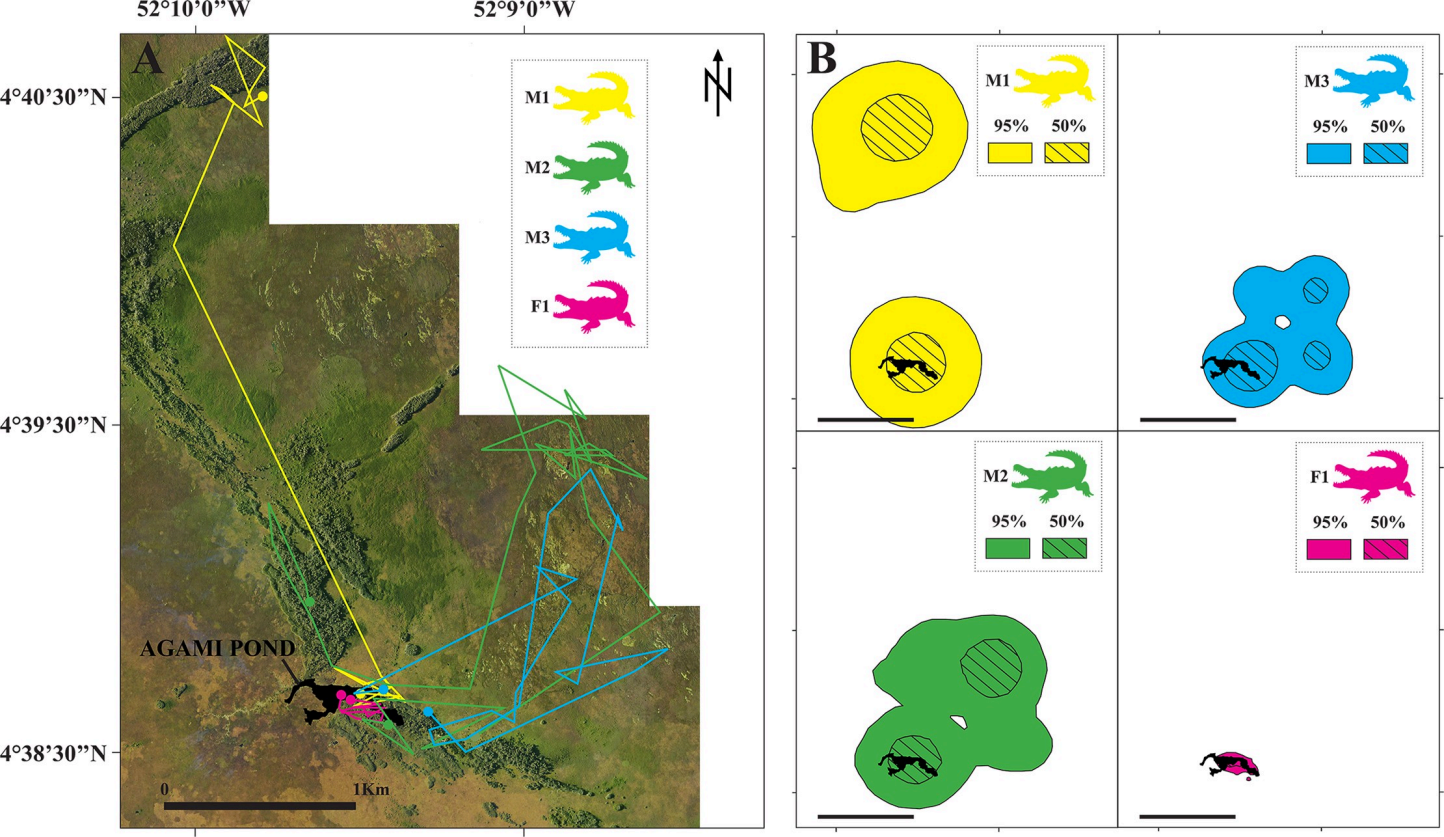
Movement patterns of the four black caimans fitted with Argos satellite transmitters. (A) Map and recorded locations of the black caimans (F1, M1, M2, M3); (B: the scale half of A) KUD 50% (solid color) and KUD 95% (hatched color) calculated from the location data.

Crocodilians are a particularly understudied group, despite the role they play in wetland ecosystems as top generalist predators. To date, satellite tracking in crocodilians has only been carried out in the estuarine crocodile (*Crocodylus porosus*, [46,47,57–59]). Given our observations during the first two sampling periods (D_1_: October 2013 and W_2_: May 2014), we hypothesized that there would be major differences in the black caiman’s movement patterns across seasons due to differences in prey availability and/or access to breeding and nesting areas (whose precise locations remain unknown). So, to develop and implement feasible conservation plans for top predators, it is important to understand their home range dynamics. Black caimans are generally considered to be territorial, with dominant males excluding conspecifics from their home ranges [60]. This behavior by larger crocodilians could reflect competition for food, mates, and high-quality nesting sites. Access to these resources could directly affect both sexes. In Agami Pond, we observed that five larger adults (>2.5 meters) lived in distinct areas in close proximity to one another within the inundated forest; four of these caimans were captured. The movement patterns we observed using GPS and KUD home range analysis revealed strong evidence of territorial patrolling [52]. KUD home range sizes varied between 336 m^2^ and 21,511 m^2^, depending on the individual and time of year (Table 1, Fig 6). We identified three different periods based on caiman movements. During the first period, from April to June, all four caimans were found in the pond and displayed very restricted movements, confirming our visual observations of territoriality within the inundated forest. During the second period, which began after June, the caimans’ monthly movements shifted, revealing the existence of two distinct behaviors. The only female to be tagged (F1, Fig 6) remained within well-defined zones of the pond. It would seem that she explored the pond when the males were absent, although the accuracy of our satellite readings was sometimes low (i.e., nearly equal to pond size). In contrast, the three males (M1, M2, M3) exhibited nomadic behavior: they travelled in three different directions across the marshes and, within two months, had reached their maximum distances from the pond. They spent several months in these new areas (~4 km away for M1 and ~2 km away for M2 and M3; Fig 6), making short-distance movements (see KUD 50% in Fig 6). Unfortunately, we lost the signal of the most distant male (M1) after almost 8 months of transmission (December). During the third period, from March to April, the other two males (M2 and M3) returned to Agami Pond, and then we lost their signals after almost a year of transmission (Fig 6, Table 1).

Little information exists on seasonal behavior in this species, which limits our ability to interpret the preliminary tracking and home range data we obtained. In general, migration is driven by three key factors: reproduction, prey availability, and more generally, seasonal fluctuation in habitat quality. These factors may be related. It has been suggested that male and female black caimans have different microhabitat use [13], which could suggest that suitable nesting areas are heterogeneously distributed, resulting in breeding females moving greater distances. However, data on the reproduction of black caimans in French Guiana are scarce. That said, it is likely that their reproductive cycle is similar to that of other populations in the Amazon. Based on what little we know about the caiman’s mating period, it probably occurs from September to early December. In general, we would expect that male crocodilians would try to mate with as many females as possible, and, in turn, females should prefer to mate with large, dominant males [61]. Some females only mate with one male over many consecutive breeding seasons, but others mate with multiple males during the same breeding season, as was recently seen in the black caiman [62]. At the end of the dry season (October–November), females build nests using vegetation and their eggs hatch in approximately 42–90 days. Hatching coincides with the beginning of the wet season (February–March), when the newly flooded marshes provide ideal habitat for the young. Females provide parental care for several months, but baby caimans are largely independent, and most do not survive to maturity.

If we consider these reproductive patterns in tandem with our tracking data, several hypotheses suggest themselves. First, Agami Pond does not appear to be a major nesting site, as we only observed and captured three juveniles there during the wet season (W_2_, W_3_). Caiman abundance in the pond was quite high, which increases the predation risk faced by juveniles. Also, the adults that we observed did not seem to display mating behavior. Consequently, it seems that the seasonal presence of water bird colonies, and especially the Agami heron colony, was the factor drawing reproductive adults to the pond. Starting in the month of June, which is when the Agami herons left the pond [3], the adult male caimans also left the pond, possibly to breed with females in more suitable areas and/or areas with more resources. In contrast, the site fidelity displayed by the one female (F1) that we tagged was more complicated to interpret. Indeed, during the dry season, we only observed one adult in the pond—a male—whom we caught in the open water. Female black caimans only breed once every 2–3 years, so perhaps our lone female was laying. It would have been possible for a female to lay eggs far enough away from the pond to remain unobserved during the dry season, but close enough for one or two juveniles to recklessly wander into the pond during the wet season. In this hypothetical scenario, adult males would have left following the departure of the herons, in search of areas with higher prey availability and aided by the increase in marsh water levels. However, it was clear from our tracking data that two males returned to the pond around the same time as the herons the following year. In a six-month study of tagged *C*. *porosus*, Campbell et al. [47] observed that males exhibited two distinct behaviors: there were males that displayed site fidelity as well as nomadic males. The researchers suggested that site fidelity resulted from males patrolling territories around the female home ranges to maximize reproductive success, while the nomads were subordinate animals that were forced to travel greater distances in search of unguarded females. In contrast, the females travelled up to 54 km away from the breeding area [47]. As no data on sex-specific dispersal are available for any other Neotropical crocodilians, further studies should be performed to better understand movement patterns during the mating period.

Given the low abundance of neonates and our inability to recapture any microchipped animals in Agami Pond, we spent four nights total exploring 75 km of the Kaw River to observe and sample caimans (May 10–11, 2014 and May 12–13, 2015). We observed very few black caimans over the four days (24 individuals in total), and their age distribution was very homogeneous (i.e., complete absence of adults [>2 meters]). In three locations, however, we observed large groups of neonates (>20 individuals), which probably represented three different yearly ponds. We did not capture any black caimans that had been microchipped in Agami Pond. The results of this additional sampling effort tended to confirm our previous conclusions: along the Kaw River, the visible abundance of adult black caimans is very low, and limited reproductive activity seems exist along the river (three nests found along the 75 km surveyed). It is also unlikely that individuals could migrate from Agami Pond to the river. The most recent regional-scale genetics study showed that animals living in the black waters of the Kaw swamp (i.e., Agami Pond and the Kaw River) in French Guiana were genetically differentiated from animals living in the Approuague estuary [12].

### Management implications

Large-bodied top predators can shape communities because their interactions with species at lower trophic levels result in cascading effects on prey and non-prey [11]. The dietary shifts exhibited by crocodilians may have significant impacts on ecosystem functioning and may be a fundamental factor underlying broader changes in ecological niches [41]. The movement of different-sized alligators (*Alligator mississippiensis*) has, for example, been shown to create functional connectivity among aquatic systems [54,63]. However, it is challenging to characterize the diets of crocodilians because they are aquatic and often hunt at night.

Remote monitoring tools are especially important when studying species that are easily disturbed, difficult to observe in the wild, and able to travel large distances. Crocodilians display these traits: they are shy, cryptic, semi-aquatic animals that live in often inaccessible habitats, and they spend long periods of time submerged and/or out of sight. This study is the first to couple stable isotope analysis with satellite telemetry to explore crocodilian ecology. Satellite telemetry has been very useful for studying the movements of birds, mammals, fishes, and marine turtles [28]. Surprisingly, it has rarely been used in research on crocodilians. The same is true for stable isotope analysis, which has been used in fewer than 10 studies on the trophic ecology of crocodilians even though this taxa is an excellent candidate for its usage.

Black caiman populations declined dramatically in the mid-20th century, with the species becoming extinct or locally rare over most of its range due to habitat destruction and hunting [42]. The successful re-establishment of this species is now dependent on the conservation efforts being undertaken throughout the Amazon Basin. Although the black caiman is currently highly protected by law, populations require continuous monitoring. Because the Kaw Marsh black caiman population is not connected to the Brazilian population, the former, which is greatly isolated, is likely to be threatened by any habitat change or disturbance. For several years, other Amazonian populations have been the focus of conservation projects that aim to better understand population origin, density, distribution, and gene flow. Studies on species foraging dynamics and behavior can provide conservation biologists and wildlife managers with essential information on species’ roles within ecosystems. Such knowledge is all the more crucial given that many apex predators are in decline and being lost worldwide. Our findings on the trophic ecology and movement patterns of Agami Pond black caimans are a first step towards the better conservation of this species in French Guiana. However, further research is needed to shed light on the black caiman’s “dark side”—we still know very little about its movements, breeding sites, and nesting sites. In areas of extensive intact habitat and/or that are subject to less anthropogenic disturbance, it is important to protect the nurseries [4].

## Supporting information

S1 Table Stable isotopic values. δ^13^C and δ^15^N values for the different ecosystem compartments sampled across seasons (D1, W2, W3).
(PDF)

## References

[1] Guiral D, Rougier C. Trap size and prey selection of two coexisting bladderwort (Utricularia) species in a pristine tropical pond (French Guiana) at different trophic levels. Int J Lim. 2007; 43: 147–159.

[2] de Thoisy B, Auffret E. Possible extension of the distributional area of Black caiman in French Guiana. CSG Newsletter. 2003; 22: 17–18.

[3] Stier A, Kushlan J. Agami Heron Conservation Plan (Agamia agami). edit. Benoit Hurpeau: GEPOG Association; 2015.

[4] Ross P. Crocodiles: An Action Plan for their Conservation. IUCN, Gland, Switzerland; 1998.

[5] Thorbjarnarson JB. Black Caiman Melanosuchus niger. In Crocodiles. Status Survey and Conservation Action Plan Third Edition, ed. by Manolis S.C. and Stevenson C. Crocodile Specialist Group: Darwin; 2010 pp. 29–39.

[6] Plotkin MJ, Medem F, Mittermeier RA, Constable IA. Distribution and conservation of the black caiman (Melanosuchus niger) In: Advances in Herpetology and Evolutionary Biology. Rhodin A., Mitaya K., Eds, Cambridge, MA, Harward University Press; 1983.

[7] Rebêlo GH, Magnusson WE. An analysis of the effect of hunting on *Caiman crocodilus* and *Melanosuchus niger* base on the sizes of confiscated skins. Biol Conserv. 1983; 26: 95–104.

[8] Brazaitis P. The Caiman of the Pantanal, past, present and future. In Crocodiles, Proceedings of the 8th working Meeting of the IUCN/SSC Crocodile Specialist Group, Quito, Ecuador, 119–124 IUCN, Gland, Switzerland; 1989.

[9] Ron SR. Human influence on the wariness of *Melanosuchus niger* and *Caiman crocodilus* in Cuyabeno, Ecuador. J Herpetol. 1998; 32: 320–324.

[10] Da Silveira R, Thorbjarnarson JB. Conservation implications of commercial hunting of black and spectacled caiman in the Mamirauá Sustainable Development Reserve, Brazil. Biol Conserv. 1999; 88: 103–9.

[11] Estes JA, Terborgh J, Brashares JS, Power ME, Berger J, Bond WJ, et al Trophic downgrading of planet Earth. Science. 2011; 333: 301–306. 10.1126/science.1205106 21764740

[12] de Thoisy B, Hrbek T, Farias IP, Vasconcelos WR, Lavergne A. Genetic structure, population dynamics, and conservation of black caiman (*Melanosuchus niger*). Biol Conserv. 2006; 133: 474–482.

[13] Da Silveira R, Magnusson WE. Diets of Spectacled and Black Caiman in the Anavilhanas Archipelago, Central Amazonia, Brazil. J Herpetol. 1999; 33: 181–92.

[14] Da Silveira R, Magnusson WE, Campos Z. Monitoring the distribution, abundance and breeding areas of *Caiman crocodilus crocodilus* and *Melanosuchus niger* in the Anavilhanas Archipelago, Central Amazonia, Brazil. J Herpetol. 1997; 31: 514–520.

[15] Da Silveira R, Viana JP. Amazonian Crocodilians: a keystone species for ecology and management… or simply bait? CSG Newsletter. 2003; 22: 16–17.

[16] CITES. Convention sur le commerce international des espèces de faune et de flore sauvages menacees d’extinction. Quatorzième session de la Conférence des Parties La Haye (Pays-Bas), 3–15 juin 2007. CoP14 Prop. 13 (Rev. 1); 2007.

[17] Hoenner X, Whiting SD, Hindell MA, McMahon CR. Enhancing the Use of Argos Satellite Data for Home Range and Long Distance Migration Studies of Marine Animals. PLoS ONE. 2012; 7(7): e40713 10.1371/journal.pone.0040713 22808241 PMC3395646

[18] Rooney N, McCann K, Gellner G, Moore JC. Structural asymmetry and the stability of diverse food webs. Nature. 2006; 442: 265–269. 10.1038/nature04887 16855582

[19] McCann KS, Rasmussen JB, Umbanhowar J. The dynamics of spatially coupled food webs. Ecol Lett. 2005; 8: 513–523. 10.1111/j.1461-0248.2005.00742.x 21352455

[20] Debenay JP, Guiral D, Parra M. Behaviour and taphonomic loss in foraminiferal assemblages of mangrove swamps of French Guiana. Mar Geol. 2004; 208: 295–314.

[21] Blanc M, de Thoisy B. Black caimans (Melanosuchus niger) in the Kaw swamps natural reserve, French Guiana: A first year survey. IUCN/SSC/Crocodile Specialist Group Newsletter. 2001; 20: 30–31.

[22] Ouboter PE, Vie JC, Montford T, Blanc M, Prevoteau JM. Black caiman population in Kaw swamps. IUCN/SSC/Crocodile Specialist Group Newsletter. 1999; 19: 13–15.

[23] Post DM. Using stable isotopes to estimate trophic position: Models, methods, and assumptions. Ecology. 2002; 83:703–718.

[24] Hobson KA. Tracing origins and migration of wildlife using stable isotopes: a review. Oecol. 1999; 120: 314–326. 10.1007/s004420050865 28308009

[25] Caut S, Guirlet E, Angulo E, Das K, Girondot M. Isotope analysis reveals two feeding areas for the Atlantic leatherbactk turtles. PLoS ONE. 2008; 3(3):e1845 10.1371/journal.pone.0001845 18365003 PMC2267998

[26] Coplen TB. Guidelines and recommended terms for expression of stable-isotope-ratio and gas-ratio measurement results. Rapid Commun Mass Spectrom. 2011; 25: 2538–2560. 21910288 10.1002/rcm.5129

[27] Post DM, Layman CA, Arrington DA, Takimoto G, Quattrochi J, Montana CG. Getting to the fat of the matter: models, methods and assumptions for dealing with lipids in stable isotope analyses. Oecol. 2007; 152: 179–189. 10.1007/s00442-006-0630-x 17225157

[28] Dalerum F, Angerbjörn A. Resolving temporal variation in vertebrate diets using naturally occurring stable isotopes. Oecol. 2005; 144: 647–658. 10.1007/s00442-005-0118-0 16041545

[29] Caut S. Isotope incorporation in broad-snouted caimans (crocodilians). Biol Open. 2013; 2: 629–634. 10.1242/bio.20134945 23789113 PMC3683165

[30] Rosenblatt AE, Heithaus MR. Slow isotope turnover rates and low discrimination values in the American alligator: implications for interpretation of ectotherm stable isotope data. Physiol Biochem Zool. 2013; 86: 137 10.1086/668295 23303328

[31] Caut S, Angulo E, Courchamp F. Variation in discrimination factors (Δ^15^N and Δ^13^C): the effect of diet isotopic values and applications for diet reconstruction. J Appl Ecol. 2009; 46: 443–453.

[32] Caut S, Angulo E, Courchamp F. Caution on isotopic model use for analyses of consumer diet. Can J Zool. 2008; 86: 438–445.

[33] Parnell AC, Inger R, Bearhop S, Jackson AL. Source partitioning using stable isotopes: coping with too much variation. Plos ONE. 2010; 5(3): e9672 10.1371/journal.pone.0009672 20300637 PMC2837382

[34] Bond AL, Diamond AW. Recent bayesian stable-isotope mixing models are highly sensitive to variation in discrimination factors. Ecol Appl. 2011; 21: 1017–1023. 21774408 10.1890/09-2409.1

[35] Ward EJ, Semmens BX, Phillips DL, Moore JW, Bouwes N. A quantitative approach to combine sources in stable isotope mixing models. Ecosphere. 2011; 2:art19.

[36] Argos User’s Manual. Available online at http://www.argos-system.org/manual/ (accessed 16 May 2018); 2016.

[37] Aguilera X, Van Damme PA, Coronel JS, Oberdorff T. Distribution patterns, population status and conservation of *Melanosuchus niger* and *Caiman yacare* (Crocodylia, Alligatoridae) in oxbow lakes of the Ichilo River floodplain, Bolivia. Rev Biol Trop. 2008; 56: 909–929. 19256453 10.15517/rbt.v56i2.5633

[38] Worton BJ. Kernel methods for estimating the utilization distribution in home-range studies. Ecology. 1989; 70: 164–168.

[39] Calenge A. The package adehabitat for the R software: tool for the analysis of space and habitat use by animals. Ecol model. 2006; 197: 1035.

[40] R Development Core Team. R: A language and environment for statistical computing. R Foundation for Statistical Computing Vienna, Austria: ISBN 3-900051-07-0. Available: http://www.R-project.org. Accessed 2012 Jun 4; 2010.

[41] Radloff FGT, Hobson KA, Leslie AJ. Characterising ontogenetic niche shifts in Nile crocodile using stable isotope (δ^13^C and δ^15^N) analyses of scute keratin. Isotopes Environ Health Stud. 2012; 48, 439–456. 10.1080/10256016.2012.667808 22462522

[42] Laverty TM, Dobson AP. Dietary Overlap between Black Caimans and Spectacled Caimans in the Peruvian Amazon. Herpetol. 2013; 69: 91–101.

[43] Marques TS, Lara NR, Bassetti LA, Piña CI, Camargo PB, Verdade LM. Intraspecific isotopic niche variation in broad-snouted caiman (Caiman latirostris). Isotopes Environ Health Stud. 2013; 49: 325–35. 10.1080/10256016.2013.835309 24117429

[44] Horna JV, Cintra R, Ruesta PV. Feeding ecology of black caiman *Melanosuchus niger* in a western Amazonian forest: the effects of ontogeny and seasonality on diet composition. Ecotropica. 2001; 7: 1–11.

[45] Huchzermeyer FW. Crocodiles: Biology, Husbandry And Diseases. Cambridge, MA: CABI Publishing; 2003.

[46] Read MA, Grigg GC, Irwin SR, Shanahan D, Franklin CE. Satellite Tracking Reveals Long Distance Coastal Travel and Homing by Translocated Estuarine Crocodiles, *Crocodylus porosus*. PLoS ONE. 2007; 2(9): e949 10.1371/journal.pone.0000949 17895990 PMC1978533

[47] Campbell HA, Dwyer RG, Irwin TR, Franklin CE. Home Range Utilisation and Long-Range Movement of Estuarine Crocodiles during the Breeding and Nesting Season. PLoS ONE. 2013; 8(5): e62127 10.1371/journal.pone.0062127 23650510 PMC3641080

[48] Wallace KM, Leslie AJ. Diet of the Nile crocodile, *Crocodylus niloticus*, in the Okavango Delta, Botswana. J Herpetol. 2008; 42:361–368.

[49] Villamarín F, Jardine TD, Bunn SE, Marioni B, Magnusson WE. Body size is more important than diet in determining stable-isotope estimates of trophic position in crocodilians. Scientific Reports. 2018; 8:2020 10.1038/s41598-018-19918-6 29386654 PMC5792559

[50] Magnusson WE, da Silva EV, Lima AP. Diets of Amazonian Crocodilians. J Herpethol. 1987; 21: 85–95.

[51] Wheatley PV, Peckham H, Newsome SD, Koch PL. Estimating marine resource use by the American crocodile *Crocodylus acutus* in southern Florida, USA. Mar Ecol Prog Ser. 2012; 447, 211–229.

[52] Woodborne S, Huchzermeyer KDA, Govender D, Pienaar DJ, Hall G, Myburgh JG, et al Ecosystem change and the Olifants River crocodile mass mortality events. Ecosphere. 2012; 3(10): 87

[53] Boggs ASP, Hamlin HJ, Nifong JC, Kassim BL, Lowers RH, Galligan TM, et al Urinary iodine and stable isotope analysis to examine habitat influences on thyroid hormones among coastal dwelling American alligators. Gen Comp Endocrinol. 2016; 226: 5–13. 10.1016/j.ygcen.2015.12.006 26684734 PMC4778256

[54] Rosenblatt AE, Heithaus MR. Does variation in movement tactics and trophic interactions among American alligators create habitat linkages? J Anim Ecol. 2011; 80: 786–798. 10.1111/j.1365-2656.2011.01830.x 21418209

[55] Villamarín F, Jardine TD, Bunn SE, Marioni B, Magnusson WE. Opportunistic top predators partition food resources in a tropical freshwater ecosystem. Freshwater Biology. 2017; 1–12.

[56] Guiral D, Le Guen R. Guyane Oceane. IRD Editions/Le Guen; 2012.

[57] Kay WR. Movement and ranges of radio-tracked Crocodylus porosus in the Cambridge Gulf region of Western Australia. Wildlife Res. 2004; 31, 495–508.

[58] Thomas B, Holland JD, Minot EO. Home range and movement patterns of an Estuarine Crocodile *Crocodylus porosus*: a satellite tracking pilot study. Northern Territory Naturalist. 2010; 22: 60–74

[59] Baker JB, Franklin CE, Campbell HA, Irwin TR, Dwyer RG. Ontogenetic shifts in the nesting behaviour of female crocodiles. Oecologia. 2019; 189: 891–904. 10.1007/s00442-019-04382-4 30868373

[60] Da Silveira R. Conservação e manejo do jacaré açu (*Melanosuchus niger*) na Amazônia brasileira Verdade L.M., Larriera A. (Eds.), Conservação e Manejo de Jacarés e Crocodilos da América Latina–La Conservación y el Manejo de Caimanes y Cocodrilos de América Latina, CN Editora, Piracicaba, São Paulo (2002), pp. 61–78; 2002.

[61] Garrick LD, Lang JW. Social Signals and Behaviors of Adult Alligators and Crocodiles. Integrative and Comparative Biology. 1977; 17:225–239.

[62] Muniz FL, Da Silveira R, Campos Z, Magnusson WE, Hrbek T, Farias IP. Multiple paternity in the Black Caiman (*Melanosuchus niger*) population in the Anavilhanas National Park, Brazilian Amazonia. Amphibia-Reptilia. 2011; 32: 428–434.

[63] Subalusky AL, Fitzgerald LA, Smith LL. Ontogenetic niche shifts in the American Alligator establish functional connectivity between aquatic systems. Biol Conserv. 2009; 142: 1507–1514.

