# Supplementary material for: The dark side of the black caiman: Shedding light on species dietary ecology and movement in Agami Pond, French Guiana
Source: PLoS One. 2019 Jun 24;14(6):e0217239. doi: 10.1371/journal.pone.0217239 (PMC6590786; doi:10.1371/journal.pone.0217239)
Supplement: S1 Table — δ13C and δ15N values for the different ecosystem compartments sampled across seasons (D1, W2, W3). (PDF) [file pone.0217239.s001.pdf]

S1 Table. Stable isotopic values. $\delta^{13}C$ and $\delta^{15}N$ values for the different ecosystem compartments sampled across seasons (D1, W2, W3).

SEASON	COMPARTMENT	REF	SPECIES	$\delta^{13}C$	$\delta^{15}N$	SIZE (cm)
D1	CAIMAN	C1	*Melanosuchus niger*	-26,7	5,0	65
D1	CAIMAN	C2	*Melanosuchus niger*	-28,8	5,2	70
D1	CAIMAN	C3	*Melanosuchus niger*	-27,2	5,2	87
D1	CAIMAN	C4	*Melanosuchus niger*	-27,9	5,4	92
D1	CAIMAN	C5	*Melanosuchus niger*	-27,8	6,3	99
D1	CAIMAN	C6	*Melanosuchus niger*	-26,8	6,1	114
D1	CAIMAN	C7	*Melanosuchus niger*	-27,0	6,3	118
D1	CAIMAN	C8	*Melanosuchus niger*	-26,6	6,3	122
D1	CAIMAN	C9	*Melanosuchus niger*	-27,9	6,8	123
D1	CAIMAN	C10	*Melanosuchus niger*	-26,9	6,8	126
D1	CAIMAN	C11	*Melanosuchus niger*	-27,4	7,0	132
D1	CAIMAN	C12	*Melanosuchus niger*	-27,2	7,2	136
D1	CAIMAN	C13	*Melanosuchus niger*	-27,0	6,8	139
D1	CAIMAN	C14	*Melanosuchus niger*	-26,7	7,1	145
D1	CAIMAN	C15	*Melanosuchus niger*	-26,5	6,7	147
D1	CAIMAN	C16	*Melanosuchus niger*	-25,8	7,6	149
D1	CAIMAN	C17	*Melanosuchus niger*	-26,3	7,6	150
D1	CAIMAN	C19	*Melanosuchus niger*	-27,3	6,8	155
D1	CAIMAN	C20	*Melanosuchus niger*	-27,2	7,6	159
D1	CAIMAN	C21	*Melanosuchus niger*	-27,0	7,2	165
D1	CAIMAN	C22	*Melanosuchus niger*	-27,1	7,4	172
D1	CAIMAN	C23	*Melanosuchus niger*	-27,5	6,9	174
D1	CAIMAN	C24	*Melanosuchus niger*	-26,3	7,4	175
D1	CAIMAN	C25	*Melanosuchus niger*	-27,1	7,4	185
D1	CAIMAN	C26	*Melanosuchus niger*	-27,8	7,5	278
D1	CAIMAN	C1	*Melanosuchus niger*	-28,0	3,3	65

D1	CAIMAN	C2	*Melanosuchus niger*	-28,5	3,9	70
D1	CAIMAN	C3	*Melanosuchus niger*	-29,7	3,8	87
D1	CAIMAN	C4	*Melanosuchus niger*	-28,9	4,2	92
D1	CAIMAN	C5	*Melanosuchus niger*	-27,2	4,4	99
D1	CAIMAN	C6	*Melanosuchus niger*	-26,4	4,5	114
D1	CAIMAN	C7	*Melanosuchus niger*	-27,8	4,4	118
D1	CAIMAN	C8	*Melanosuchus niger*	-28,4	5,3	122
D1	CAIMAN	C9	*Melanosuchus niger*	-28,5	5,0	123
D1	CAIMAN	C10	*Melanosuchus niger*	-27,2	5,7	126
D1	CAIMAN	C11	*Melanosuchus niger*	-27,5	5,7	132
D1	CAIMAN	C12	*Melanosuchus niger*	-27,9	5,8	136
D1	CAIMAN	C13	*Melanosuchus niger*	-27,1	5,5	139
D1	CAIMAN	C14	*Melanosuchus niger*	-27,1	5,9	145
D1	CAIMAN	C15	*Melanosuchus niger*	-26,8	5,2	147
D1	CAIMAN	C16	*Melanosuchus niger*	-26,0	6,3	149
D1	CAIMAN	C17	*Melanosuchus niger*	-28,2	6,3	150
D1	CAIMAN	C18	*Melanosuchus niger*	-27,8	6,0	155
D1	CAIMAN	C19	*Melanosuchus niger*	-28,1	5,8	155
D1	CAIMAN	C20	*Melanosuchus niger*	-27,5	6,2	159
D1	CAIMAN	C21	*Melanosuchus niger*	-27,7	5,7	165
D1	CAIMAN	C22	*Melanosuchus niger*	-27,3	5,9	172
D1	CAIMAN	C23	*Melanosuchus niger*	-27,2	5,1	174
D1	CAIMAN	C24	*Melanosuchus niger*	-27,0	5,9	175
D1	CAIMAN	C25	*Melanosuchus niger*	-27,3	6,2	185
D1	CAIMAN	C26	*Melanosuchus niger*	-28,7	6,5	278
D1	CAIMAN	C1	*Melanosuchus niger*	-27,2	4,5	65
D1	CAIMAN	C2	*Melanosuchus niger*	-28,0	5,1	70
D1	CAIMAN	C3	*Melanosuchus niger*	-28,8	5,5	87
D1	CAIMAN	C4	*Melanosuchus niger*	-28,0	5,9	92
D1	CAIMAN	C5	*Melanosuchus niger*	-26,9	6,2	99

D1	CAIMAN	C6	*Melanosuchus niger*	-26,5	6,3	114
D1	CAIMAN	C7	*Melanosuchus niger*	-27,3	6,5	118
D1	CAIMAN	C8	*Melanosuchus niger*	-28,3	6,8	122
D1	CAIMAN	C9	*Melanosuchus niger*	-28,2	6,8	123
D1	CAIMAN	C10	*Melanosuchus niger*	-27,0	7,1	126
D1	CAIMAN	C11	*Melanosuchus niger*	-27,3	7,4	132
D1	CAIMAN	C12	*Melanosuchus niger*	-27,4	7,1	136
D1	CAIMAN	C13	*Melanosuchus niger*	-27,2	7,4	139
D1	CAIMAN	C14	*Melanosuchus niger*	-26,7	7,2	145
D1	CAIMAN	C15	*Melanosuchus niger*	-26,3	6,7	147
D1	CAIMAN	C16	*Melanosuchus niger*	-26,2	7,4	149
D1	CAIMAN	C17	*Melanosuchus niger*	-27,8	7,2	150
D1	CAIMAN	C18	*Melanosuchus niger*	-27,1	7,4	155
D1	CAIMAN	C19	*Melanosuchus niger*	-27,6	6,9	155
D1	CAIMAN	C20	*Melanosuchus niger*	-27,7	7,2	159
D1	CAIMAN	C21	*Melanosuchus niger*	-27,2	7,2	165
D1	CAIMAN	C22	*Melanosuchus niger*	-27,2	7,2	172
D1	CAIMAN	C23	*Melanosuchus niger*	-27,9	7,2	174
D1	CAIMAN	C24	*Melanosuchus niger*	-26,8	7,4	175
D1	CAIMAN	C25	*Melanosuchus niger*	-26,9	7,9	185
D1	CAIMAN	C26	*Melanosuchus niger*	-28,8	7,5	278
D1	FISH	F4	*Chaetobranchus flavescens*	-27,5	6,6	
D1	FISH	F4	*Chaetobranchus flavescens*	-28,0	5,1	
D1	FISH	F4	*Chaetobranchus flavescens*	-28,7	6,4	
D1	FISH	F1	*Hemigrammus sp*	-31,0	5,2	
D1	FISH	F1	*Hemigrammus sp*	-30,6	4,5	
D1	FISH	F1	*Hemigrammus sp*	-30,8	4,9	
D1	FISH	F1	*Hemigrammus sp*	-30,3	5,1	
D1	FISH	F6	*Hoplerythrinus unitaeniatus*	-27,5	7,4	
D1	FISH	F6	*Hoplerythrinus unitaeniatus*	-26,7	9,3	

D1	FISH	F5	*Hoplias malabaricus*	-29,0	7,7	
D1	FISH	F5	*Hoplias malabaricus*	-28,0	8,3	
D1	FISH	F5	*Hoplias malabaricus*	-26,8	8,8	
D1	FISH	F3	*Metynnis lippincottianus*	-30,7	4,1	
D1	FISH	F2	*Pristella maxillaris*	-29,4	5,4	
D1	FISH	F2	*Pristella maxillaris*	-28,5	5,2	
D1	FISH	F2	*Pristella maxillaris*	-28,7	5,6	
D1	FISH	F2	*Pristella maxillaris*	-26,9	6,4	
D1	INVERTEBRATE	I1	Aranea	-26,9	4,2	
D1	INVERTEBRATE	I6	Coleoptera	-29,2	3,7	
D1	INVERTEBRATE	I2	Diptera	-27,1	3,8	
D1	INVERTEBRATE	I2	Diptera	-26,5	3,3	
D1	INVERTEBRATE	I5	Hymenoptera	-25,7	4,8	
D1	INVERTEBRATE	I4	Odonata	-27,7	3,8	
D1	INVERTEBRATE	I3	Orthoptera	-26,1	4,2	
D1	INVERTEBRATE	I3	Orthoptera	-25,7	3,9	
D1	PLANKTON	P1		-29,2	-0,4	
D1	PLANKTON	P1		-29,2	0,7	
D1	PLANKTON	P1		-31,1	0,3	
D1	PLANKTON	P1		-31,3	0,8	
D1	PLANKTON	P1		-31,2	0,6	
D1	PLANT	PL6	*Chrysobalanus icaco*	-29,3	2,2	
D1	PLANT	PL8	grand carex	-29,7	0,2	
D1	PLANT	PL9	*Hydrocotyle umbellata*	-30,6	-2,2	
D1	PLANT	PL4	*Irlbachia alata*	-29,5	0,2	
D1	PLANT	PL10	mini graminée	-29,8	-0,2	
D1	PLANT	PL1	*Nymphaea rudgeana*	-26,5	0,0	
D1	PLANT	PL2	*Pterocarpus officinales*	-27,7	0,3	
D1	PLANT	PL5	*Sagittaria lancifolia*	-29,0	0,6	
D1	PLANT	PL3	*Thelypteris interrupta*	-29,1	-1,7	

D1	PLANT	PL7	*Utricularia hydrocarpa*	-31,1	1,7	
D1	SHRIMP	S1	*Macrobrachium jelskii*	-30,3	5,6	
D1	SHRIMP	S1	*Macrobrachium jelskii*	-30,6	7,1	
D1	SHRIMP	S1	*Macrobrachium jelskii*	-29,5	4,6	
D1	SHRIMP	S1	*Macrobrachium jelskii*	-29,4	7,1	
D1	SHRIMP	S1	*Macrobrachium jelskii*	-29,9	7,0	
W2	AMPHIBIAN	A1	*Pipa snethlageae*	-29,5	8,0	
W2	AMPHIBIAN	A1	*Pipa snethlageae*	-28,2	8,5	
W2	BIRD	O1	*Agamia agami*	-29,5	7,3	
W2	BIRD	O1	*Agamia agami*	-26,2	4,7	
W2	BIRD	O1	*Agamia agami*	-26,2	4,5	
W2	BIRD	O1	*Agamia agami*	-30,2	7,2	
W2	BIRD	O1	*Agamia agami*	-28,8	7,0	
W2	BIRD	O1	*Agamia agami*	-29,8	9,1	
W2	BIRD	O1	*Agamia agami*	-27,9	8,1	
W2	BIRD	O1	*Agamia agami*	-28,1	8,9	
W2	BIRD	O1	*Agamia agami*	-27,8	8,3	
W2	BIRD	O1	*Agamia agami*	-29,1	8,1	
W2	BIRD	O1	*Agamia agami*	-28,7	7,7	
W2	BIRD	O1	*Agamia agami*	-29,0	7,4	
W2	BIRD	O1	*Agamia agami*	-29,3	7,7	
W2	BIRD	O1	*Agamia agami*	-29,7	7,6	
W2	BIRD	O1	*Agamia agami*	-28,5	9,1	
W2	BIRD	O1	*Agamia agami*	-27,9	7,1	
W2	BIRD	O1	*Agamia agami*	-29,9	7,4	
W2	BIRD	O1	*Agamia agami*	-30,2	6,9	
W2	CAIMAN	C18	*Melanosuchus niger*	-25,6	7,3	155
W2	CAIMAN	C27	*Melanosuchus niger*	-26,5	5,3	46
W2	CAIMAN	C28	*Melanosuchus niger*	-27,6	4,9	50
W2	CAIMAN	C29	*Melanosuchus niger*	-27,7	6,3	68

W2	CAIMAN	C30	*Melanosuchus niger*	-28,0	4,7	83
W2	CAIMAN	C31	*Melanosuchus niger*	-27,2	5,0	83
W2	CAIMAN	C32	*Melanosuchus niger*	-28,4	5,1	86
W2	CAIMAN	C33	*Melanosuchus niger*	-27,8	6,2	88
W2	CAIMAN	C34	*Melanosuchus niger*	-27,7	6,2	89
W2	CAIMAN	C35	*Melanosuchus niger*	-27,5	6,6	108
W2	CAIMAN	C36	*Melanosuchus niger*	-26,3	6,5	126
W2	CAIMAN	C37	*Melanosuchus niger*	-26,1	7,5	128
W2	CAIMAN	C38	*Melanosuchus niger*	-27,0	6,5	132
W2	CAIMAN	C39	*Melanosuchus niger*	-26,2	6,4	135
W2	CAIMAN	C40	*Melanosuchus niger*	-27,3	7,0	140
W2	CAIMAN	C41	*Melanosuchus niger*	-27,4	6,8	141
W2	CAIMAN	C42	*Melanosuchus niger*	-24,8	6,9	142
W2	CAIMAN	C43	*Melanosuchus niger*	-27,9	7,0	154
W2	CAIMAN	C44	*Melanosuchus niger*	-25,7	7,1	158
W2	CAIMAN	C45	*Melanosuchus niger*	-28,7	7,0	160
W2	CAIMAN	C46	*Melanosuchus niger*	-28,1	7,2	183
W2	CAIMAN	C47	*Melanosuchus niger*	-26,6	7,4	196
W2	CAIMAN	C48	*Melanosuchus niger*	-28,1	7,4	210
W2	CAIMAN	C49	*Melanosuchus niger*	-28,3	7,1	214
W2	CAIMAN	C50	*Melanosuchus niger*	-27,9	7,2	216
W2	CAIMAN	C51	*Melanosuchus niger*	-27,2	7,3	220
W2	CAIMAN	C52	*Melanosuchus niger*	-27,2	7,1	221
W2	CAIMAN	C53	*Melanosuchus niger*	-27,5	7,2	240
W2	CAIMAN	C54	*Melanosuchus niger*	-27,6	7,4	326
W2	CAIMAN	C27	*Melanosuchus niger*	-27,2	4,8	46
W2	CAIMAN	C28	*Melanosuchus niger*	-27,7	3,6	50
W2	CAIMAN	C29	*Melanosuchus niger*	-28,2	4,5	68
W2	CAIMAN	C30	*Melanosuchus niger*	-27,6	2,6	83
W2	CAIMAN	C31	*Melanosuchus niger*	-27,4	3,4	83

W2	CAIMAN	C32	*Melanosuchus niger*	-28,3	3,1	86
W2	CAIMAN	C33	*Melanosuchus niger*	-27,5	6,3	88
W2	CAIMAN	C34	*Melanosuchus niger*	-27,3	4,1	89
W2	CAIMAN	C35	*Melanosuchus niger*	-27,8	5,6	108
W2	CAIMAN	C36	*Melanosuchus niger*	-28,2	5,6	126
W2	CAIMAN	C37	*Melanosuchus niger*	-26,5	5,1	128
W2	CAIMAN	C38	*Melanosuchus niger*	-28,1	6,0	132
W2	CAIMAN	C39	*Melanosuchus niger*	-26,5	4,8	135
W2	CAIMAN	C40	*Melanosuchus niger*	-28,1	6,1	140
W2	CAIMAN	C41	*Melanosuchus niger*	-28,3	5,8	141
W2	CAIMAN	C42	*Melanosuchus niger*	-26,7	5,4	142
W2	CAIMAN	C43	*Melanosuchus niger*	-28,5	6,0	154
W2	CAIMAN	C44	*Melanosuchus niger*	-27,0	5,5	158
W2	CAIMAN	C45	*Melanosuchus niger*	-28,2	6,3	160
W2	CAIMAN	C46	*Melanosuchus niger*	-28,5	6,0	183
W2	CAIMAN	C47	*Melanosuchus niger*	-26,9	6,1	196
W2	CAIMAN	C48	*Melanosuchus niger*	-28,4	6,2	210
W2	CAIMAN	C49	*Melanosuchus niger*	-28,2	5,8	214
W2	CAIMAN	C50	*Melanosuchus niger*	-28,0	6,2	216
W2	CAIMAN	C51	*Melanosuchus niger*	-27,7	6,2	220
W2	CAIMAN	C52	*Melanosuchus niger*	-27,6	6,0	221
W2	CAIMAN	C53	*Melanosuchus niger*	-27,5	6,5	240
W2	CAIMAN	C54	*Melanosuchus niger*	-27,7	6,2	326
W2	CAIMAN	C27	*Melanosuchus niger*	-26,5	4,4	46
W2	CAIMAN	C28	*Melanosuchus niger*	-28,2	4,6	50
W2	CAIMAN	C29	*Melanosuchus niger*	-27,9	6,1	68
W2	CAIMAN	C30	*Melanosuchus niger*	-26,8	3,3	83
W2	CAIMAN	C31	*Melanosuchus niger*	-27,2	4,8	83
W2	CAIMAN	C32	*Melanosuchus niger*	-27,5	4,3	86
W2	CAIMAN	C33	*Melanosuchus niger*	-27,6	6,7	88

W2	CAIMAN	C34	*Melanosuchus niger*	-27,1	5,5	89
W2	CAIMAN	C35	*Melanosuchus niger*	-27,3	6,7	108
W2	CAIMAN	C36	*Melanosuchus niger*	-27,5	6,6	126
W2	CAIMAN	C37	*Melanosuchus niger*	-25,9	6,9	128
W2	CAIMAN	C38	*Melanosuchus niger*	-27,7	6,8	132
W2	CAIMAN	C39	*Melanosuchus niger*	-26,4	6,3	135
W2	CAIMAN	C40	*Melanosuchus niger*	-27,6	7,7	140
W2	CAIMAN	C41	*Melanosuchus niger*	-27,2	7,1	141
W2	CAIMAN	C42	*Melanosuchus niger*	-26,5	6,7	142
W2	CAIMAN	C43	*Melanosuchus niger*	-27,3	7,2	154
W2	CAIMAN	C44	*Melanosuchus niger*	-26,9	6,8	158
W2	CAIMAN	C45	*Melanosuchus niger*	-27,6	7,3	160
W2	CAIMAN	C46	*Melanosuchus niger*	-28,0	7,1	183
W2	CAIMAN	C47	*Melanosuchus niger*	-26,7	7,4	196
W2	CAIMAN	C48	*Melanosuchus niger*	-28,0	7,5	210
W2	CAIMAN	C49	*Melanosuchus niger*	-27,9	7,1	214
W2	CAIMAN	C50	*Melanosuchus niger*	-27,6	7,1	216
W2	CAIMAN	C51	*Melanosuchus niger*	-27,2	7,5	220
W2	CAIMAN	C52	*Melanosuchus niger*	-27,3	7,0	221
W2	CAIMAN	C53	*Melanosuchus niger*	-27,7	7,5	240
W2	CAIMAN	C54	*Melanosuchus niger*	-27,1	7,5	326
W2	FISH	F4	*Chaetobranchus flavescens*	-28,9	6,5	
W2	FISH	F4	*Chaetobranchus flavescens*	-28,7	6,8	
W2	FISH	F1	*Hemigrammus sp*	-29,3	6,6	
W2	FISH	F1	*Hemigrammus sp*	-25,6	6,6	
W2	FISH	F1	*Hemigrammus sp*	-28,4	5,4	
W2	FISH	F1	*Hemigrammus sp*	-32,1	5,8	
W2	FISH	F6	*Hoplerythrinus unitaeniatus*	-26,3	8,7	
W2	FISH	F6	*Hoplerythrinus unitaeniatus*	-28,1	10,5	
W2	FISH	F5	*Hoplias malabaricus*	-28,0	9,2	

W2	FISH	F5	*Hoplias malabaricus*	-28,0	9,7	
W2	FISH	F5	*Hoplias malabaricus*	-28,8	7,7	
W2	FISH	F3	*Metynnis lippincottianus*	-31,6	2,7	
W2	FISH	F2	*Pristella maxillaris*	-31,0	6,8	
W2	FISH	F2	*Pristella maxillaris*	-30,7	5,5	
W2	FISH	F2	*Pristella maxillaris*	-29,3	4,3	
W2	FISH	F2	*Pristella maxillaris*	-28,7	6,9	
W2	INVERTEBRATE	I1	Aranea	-26,6	4,3	
W2	INVERTEBRATE	I6	Coleoptera	-29,2	3,4	
W2	INVERTEBRATE	I2	Diptera	-25,9	3,8	
W2	INVERTEBRATE	I5	Hymenoptera	-26,3	4,0	
W2	INVERTEBRATE	I4	Odonata	-30,3	4,2	
W2	INVERTEBRATE	I4	Odonata	-27,6	3,6	
W2	INVERTEBRATE	I3	Orthoptera	-26,2	4,0	
W2	INVERTEBRATE	I3	Orthoptera	-26,1	1,6	
W2	PLANKTON	P1		-29,7	1,4	
W2	PLANKTON	P1		-30,7	1,1	
W2	PLANKTON	P1		-30,0	-0,4	
W2	PLANKTON	P1		-30,0	0,1	
W2	PLANKTON	P1		-30,1	0,3	
W2	PLANT	PL6	*Chrysobalanus icaco*	-29,3	1,9	
W2	PLANT	PL8	grand carex	-30,2	0,8	
W2	PLANT	PL9	*Hydrocotyle umbellata*	-30,8	-2,8	
W2	PLANT	PL4	*Irlbachia alata*	-29,9	0,8	
W2	PLANT	PL10	mini graminée	-29,2	-0,3	
W2	PLANT	PL1	*Nymphaea rudgeana*	-26,1	-0,1	
W2	PLANT	PL2	*Pterocarpus officinales*	-26,7	0,3	
W2	PLANT	PL5	*Sagittaria lancifolia*	-29,7	0,6	
W2	PLANT	PL3	*Thelypteris interrupta*	-28,2	-1,3	
W2	PLANT	PL7	*Utricularia hydrocarpa*	-30,5	1,7	

W2	SHRIMP	S1	*Macrobrachium jelskii*	-29,3	5,1	
W2	SHRIMP	S1	*Macrobrachium jelskii*	-29,2	6,0	
W2	SHRIMP	S1	*Macrobrachium jelskii*	-30,4	5,9	
W2	SHRIMP	S1	*Macrobrachium jelskii*	-29,8	6,1	
W2	SHRIMP	S1	*Macrobrachium jelskii*	-30,4	6,2	
W2	SHRIMP	S1	*Macrobrachium jelskii*	-28,5	5,3	
W3	AMPHIBIAN	A1	*Pipa snethlageae*	-28,8	8,1	
W3	AMPHIBIAN	A1	*Pipa snethlageae*	-29,4	9,0	
W3	BIRD	O1	*Agamia agami*	-30,0	7,9	
W3	BIRD	O1	*Agamia agami*	-28,6	7,3	
W3	BIRD	O1	*Agamia agami*	-29,7	7,5	
W3	BIRD	O1	*Agamia agami*	-29,9	7,7	
W3	BIRD	O1	*Agamia agami*	-28,7	7,6	
W3	BIRD	O1	*Agamia agami*	-28,8	7,3	
W3	BIRD	O1	*Agamia agami*	-28,5	7,7	
W3	BIRD	O1	*Agamia agami*	-28,8	8,3	
W3	BIRD	O1	*Agamia agami*	-28,7	7,7	
W3	BIRD	O1	*Agamia agami*	-28,7	8,1	
W3	CAIMAN	C55	*Melanosuchus niger*	-29,5	5,2	60
W3	CAIMAN	C56	*Melanosuchus niger*	-26,8	5,8	77
W3	CAIMAN	C57	*Melanosuchus niger*	-27,2	4,6	83
W3	CAIMAN	C58	*Melanosuchus niger*	-27,1	4,1	86
W3	CAIMAN	C59	*Melanosuchus niger*	-27,0	4,8	90
W3	CAIMAN	C60	*Melanosuchus niger*	-27,3	5,3	95
W3	CAIMAN	C61	*Melanosuchus niger*	-27,1	5,4	97
W3	CAIMAN	C62	*Melanosuchus niger*	-27,4	6,0	102
W3	CAIMAN	C63	*Melanosuchus niger*	-28,4	6,7	108
W3	CAIMAN	C64	*Melanosuchus niger*	-27,2	6,1	109
W3	CAIMAN	C65	*Melanosuchus niger*	-27,2	6,2	111
W3	CAIMAN	C66	*Melanosuchus niger*	-27,1	6,2	112

W3	CAIMAN	C67	*Melanosuchus niger*	-27,1	5,4	115
W3	CAIMAN	C68	*Melanosuchus niger*	-27,3	7,2	190
W3	CAIMAN	C69	*Melanosuchus niger*	-28,2	7,2	193
W3	CAIMAN	C70	*Melanosuchus niger*	-26,3	7,3	195
W3	CAIMAN	C71	*Melanosuchus niger*	-27,2	7,4	234
W3	CAIMAN	C72	*Melanosuchus niger*	-25,8	7,6	254
W3	CAIMAN	C73	*Melanosuchus niger*	-26,9	7,0	272
W3	CAIMAN	C74	*Melanosuchus niger*	-26,8	7,0	320
W3	CAIMAN	C75	*Melanosuchus niger*	-26,1	7,5	346
W3	CAIMAN	C55	*Melanosuchus niger*	-28,4	3,6	60
W3	CAIMAN	C56	*Melanosuchus niger*	-26,7	4,8	77
W3	CAIMAN	C57	*Melanosuchus niger*	-26,7	2,7	83
W3	CAIMAN	C58	*Melanosuchus niger*	-27,5	2,8	86
W3	CAIMAN	C59	*Melanosuchus niger*	-27,6	3,2	90
W3	CAIMAN	C60	*Melanosuchus niger*	-27,1	3,7	95
W3	CAIMAN	C61	*Melanosuchus niger*	-27,5	3,5	97
W3	CAIMAN	C62	*Melanosuchus niger*	-27,4	5,1	102
W3	CAIMAN	C63	*Melanosuchus niger*	-27,9	5,5	108
W3	CAIMAN	C64	*Melanosuchus niger*	-26,8	5,0	109
W3	CAIMAN	C65	*Melanosuchus niger*	-26,8	5,1	111
W3	CAIMAN	C66	*Melanosuchus niger*	-27,1	5,0	112
W3	CAIMAN	C67	*Melanosuchus niger*	-27,4	4,6	115
W3	CAIMAN	C68	*Melanosuchus niger*	-27,2	6,3	190
W3	CAIMAN	C69	*Melanosuchus niger*	-28,5	6,1	193
W3	CAIMAN	C70	*Melanosuchus niger*	-27,5	6,3	195
W3	CAIMAN	C71	*Melanosuchus niger*	-27,8	6,2	234
W3	CAIMAN	C72	*Melanosuchus niger*	-27,5	6,5	254
W3	CAIMAN	C73	*Melanosuchus niger*	-27,3	5,9	272
W3	CAIMAN	C74	*Melanosuchus niger*	-27,4	5,9	320
W3	CAIMAN	C75	*Melanosuchus niger*	-27,9	6,2	346

W3	CAIMAN	C55	*Melanosuchus niger*	-29,4	5,1	60
W3	CAIMAN	C56	*Melanosuchus niger*	-27,3	6,1	77
W3	CAIMAN	C57	*Melanosuchus niger*	-27,4	4,5	83
W3	CAIMAN	C58	*Melanosuchus niger*	-27,3	4,1	86
W3	CAIMAN	C59	*Melanosuchus niger*	-27,4	4,6	90
W3	CAIMAN	C60	*Melanosuchus niger*	-27,1	4,9	95
W3	CAIMAN	C61	*Melanosuchus niger*	-27,4	5,1	97
W3	CAIMAN	C62	*Melanosuchus niger*	-27,7	6,1	102
W3	CAIMAN	C63	*Melanosuchus niger*	-28,4	7,1	108
W3	CAIMAN	C64	*Melanosuchus niger*	-27,0	6,4	109
W3	CAIMAN	C65	*Melanosuchus niger*	-27,1	6,2	111
W3	CAIMAN	C66	*Melanosuchus niger*	-26,9	6,4	112
W3	CAIMAN	C67	*Melanosuchus niger*	-27,4	5,8	115
W3	CAIMAN	C68	*Melanosuchus niger*	-27,2	7,4	190
W3	CAIMAN	C69	*Melanosuchus niger*	-28,3	7,2	193
W3	CAIMAN	C70	*Melanosuchus niger*	-26,7	7,6	195
W3	CAIMAN	C71	*Melanosuchus niger*	-27,5	7,4	234
W3	CAIMAN	C72	*Melanosuchus niger*	-26,1	8,0	254
W3	CAIMAN	C73	*Melanosuchus niger*	-27,0	7,0	272
W3	CAIMAN	C74	*Melanosuchus niger*	-26,9	7,0	320
W3	CAIMAN	C75	*Melanosuchus niger*	-27,6	6,9	346
W3	FISH	F4	*Chaetobranchus flavescens*	-29,5	6,0	
W3	FISH	F4	*Chaetobranchus flavescens*	-28,3	6,4	
W3	FISH	F1	*Hemigrammus sp*	-31,3	7,6	
W3	FISH	F1	*Hemigrammus sp*	-27,9	5,2	
W3	FISH	F1	*Hemigrammus sp*	-28,4	2,2	
W3	FISH	F1	*Hemigrammus sp*	-29,4	4,2	
W3	FISH	F6	*Hoplerythrinus unitaeniatus*	-28,8	6,4	
W3	FISH	F6	*Hoplerythrinus unitaeniatus*	-26,8	8,8	
W3	FISH	F5	*Hoplias malabaricus*	-27,2	10,0	

W3	FISH	F5	*Hoplias malabaricus*	-27,9	9,6	
W3	FISH	F5	*Hoplias malabaricus*	-26,9	8,1	
W3	FISH	F3	*Metynnis lippincottianus*	-31,1	4,1	
W3	FISH	F2	*Pristella maxillaris*	-29,7	5,3	
W3	FISH	F2	*Pristella maxillaris*	-30,5	4,7	
W3	FISH	F2	*Pristella maxillaris*	-29,0	5,2	
W3	FISH	F2	*Pristella maxillaris*	-32,1	6,4	
W3	INVERTEBRATE	I6	Coleoptera	-30,7	3,2	
W3	INVERTEBRATE	I2	Diptera	-26,6	3,7	
W3	INVERTEBRATE	I2	Diptera	-26,3	3,8	
W3	INVERTEBRATE	I5	Hymenoptera	-26,3	4,0	
W3	INVERTEBRATE	I4	Odonata	-30,2	2,7	
W3	INVERTEBRATE	I3	Orthoptera	-27,7	0,5	
W3	INVERTEBRATE	I3	Orthoptera	-24,2	4,5	
W3	PLANKTON	P1		-29,5	0,8	
W3	PLANKTON	P1		-31,2	1,0	
W3	PLANKTON	P1		-31,1	0,7	
W3	PLANKTON	P1		-30,0	0,1	
W3	PLANKTON	P1		-30,1	0,3	
W3	PLANT	PL6	*Chrysobalanus icaco*	-29,5	1,8	
W3	PLANT	PL8	grand carex	-29,6	-0,5	
W3	PLANT	PL9	*Hydrocotyle umbellata*	-29,1	-1,7	
W3	PLANT	PL4	*Irlbachia alata*	-30,1	0,1	
W3	PLANT	PL10	mini graminée	-30,4	-0,5	
W3	PLANT	PL1	*Nymphaea rudgeana*	-25,5	-0,9	
W3	PLANT	PL2	*Pterocarpus officinales*	-27,1	0,6	
W3	PLANT	PL5	*Sagittaria lancifolia*	-29,4	-0,4	
W3	PLANT	PL3	*Thelypteris interrupta*	-28,3	-1,7	
W3	PLANT	PL7	*Utricularia hydrocarpa*	-28,2	1,3	
W3	SHRIMP	S1	*Macrobrachium jelskii*	-29,5	6,4	

W3	SHRIMP	S1	*Macrobrachium jelskii*	-28,8	6,8	
W3	SHRIMP	S1	*Macrobrachium jelskii*	-28,7	6,8	
W3	SHRIMP	S1	*Macrobrachium jelskii*	-28,7	6,7	
W3	SHRIMP	S1	*Macrobrachium jelskii*	-30,0	5,1	
W3	SHRIMP	S1	*Macrobrachium jelskii*	-29,1	5,9	
